# *Eggerthella lenta* disrupts the gut vascular barrier to drive colorectal cancer liver metastasis

**DOI:** 10.1038/s41421-026-00922-4

**Published:** 2026-09-22

**Authors:** Cheng Kong, Yutao Jin, Guang Liu, Yangyang Guo, Fanying Guo, Yanlei Ma

**Affiliations:** 1 Department of Colorectal Surgery, Fudan University Shanghai Cancer Center, Shanghai, China; 2 Department of Oncology, Shanghai Medical College, Fudan University, Shanghai, China; 3 Department of Endoscopy, Fudan University Shanghai Cancer Center, Shanghai, China; 4 Guangdong Hongyuan Pukang Medical Technology Co., Ltd, Guangzhou, Guangdong China

**Keywords:** Cancer microenvironment, Cell invasion

## Abstract

Distant metastasis is the leading cause of death in colorectal cancer (CRC), and the gut vascular barrier (GVB) is the first obstacle to hematogenous spread. To investigate whether GVB function is directly influenced by metastasis-associated bacteria, we analyzed two cohorts comprising 20 healthy controls, 82 non-metastatic CRC patients, and 65 patients with liver or lung metastases. Multi-omics approaches (metagenomic sequencing and single-cell RNA sequencing) and gnotobiotic mouse models were employed to examine gut microbes that are linked to GVB disruption and metastasis. Patients with CRC liver metastasis exhibited impaired GVB and bacterial colonization at metastatic sites. *Eggerthella lenta* was enriched in patients with elevated expression of plasmalemmal vesicle-associated protein-1 (PV-1), a GVB injury marker, and its abundance was correlated with metastasis and recurrence. In vitro and in vivo, *E. lenta* compromised endothelial tight junction and GVB integrity, enhancing CRC cell migration and liver metastasis. Mechanistically, *E. lenta* adhered to endothelial cells and activated endoplasmic reticulum (ER) stress via a TLR4-dependent pathway, promoting autophagy and apoptosis. The knockdown of PERK attenuated ER stress, prevented autophagy and apoptosis, and downregulated the expression of ZO-1 and Claudin-5 induced by *E. lenta*. These findings highlight the clinical potential of microbiota-targeted strategies and PERK inhibition in the treatment of *E. lenta*-associated CRC metastasis.

## Introduction

Colorectal cancer (CRC) remains a leading cause of cancer-related mortality worldwide, with metastasis being the principal determinant of poor prognosis. The liver is the most common and critical site of distant metastasis in CRC, and understanding the mechanisms that drive hepatic dissemination is a priority. Metastasis is a complex, multi-step process, and the intravasation of tumor cells across the vascular endothelial layer into the circulation is a critical early step^1^. In recent years, the gut vascular barrier (GVB), a specialized endothelial barrier situated directly beneath the intestinal epithelium, has been identified as a crucial gatekeeper^2^. It regulates the exchange of substances and cells between the intestinal lumen and the bloodstream, thereby representing a decisive checkpoint for potential metastatic spread from the primary tumor.

The integrity of the GVB is vital for preventing pathological translocation. GVB disruption, marked by increased expression of plasmalemma vesicle-associated protein-1 (PV-1), leads to enhanced vascular permeability^3^. This defect is involved not only in facilitating the entry of tumor cells into the portal circulation but also in shaping the metastatic niche. While anti-angiogenic therapy has been a cornerstone treatment strategy, its paradoxical effects — including the promotion of a hypoxic, inflammatory microenvironment that may enhance tumor invasiveness and chemoresistance — highlight the need for alternative treatment approaches^4,5^. Emerging evidence suggests that normalizing the tumor vasculature and, specifically, restoring GVB integrity, may be more effective than current treatments at suppressing metastasis, particularly to the liver^6^.

The gut microbiota, which resides in direct contact with the GVB, is a key modulator of GVB function^7,8^. The role of the microbiota in intestinal vascular remodeling and function is well established. Notably, specific pathogenic bacteria can directly compromise GVB integrity. For instance, *Salmonella typhimurium* can breach the GVB by interfering with endothelial Wnt/β-catenin signaling, promoting bacterial dissemination to the liver^9^. Most compellingly, a recent study identified a pro-invasive *Escherichia coli* strain (C17) that induces GVB injury, facilitates bacterial translocation to the liver, and is enriched within hepatic metastases, where it co-localizes with proliferating cancer cells^10^. These findings establish a direct link between a specific microbial agent, GVB breakdown, and CRC liver metastasis (CRLM).

Despite these advances, the mechanisms by which the gut microbiota regulates GVB integrity to influence CRC metastasis, specifically to the liver, remain poorly defined. The identification of the core pathogenic bacteria responsible for GVB injury and a detailed understanding of their pro-carcinogenic mechanisms are critical knowledge gaps in this field. Therefore, this study aimed to screen for and characterize the core gut microbes associated with GVB disruption and to elucidate their specific roles and mechanisms in driving CRC liver metastasis.

## Results

### CRLM patients exhibit significant GVB impairment

**Fig. 1:**
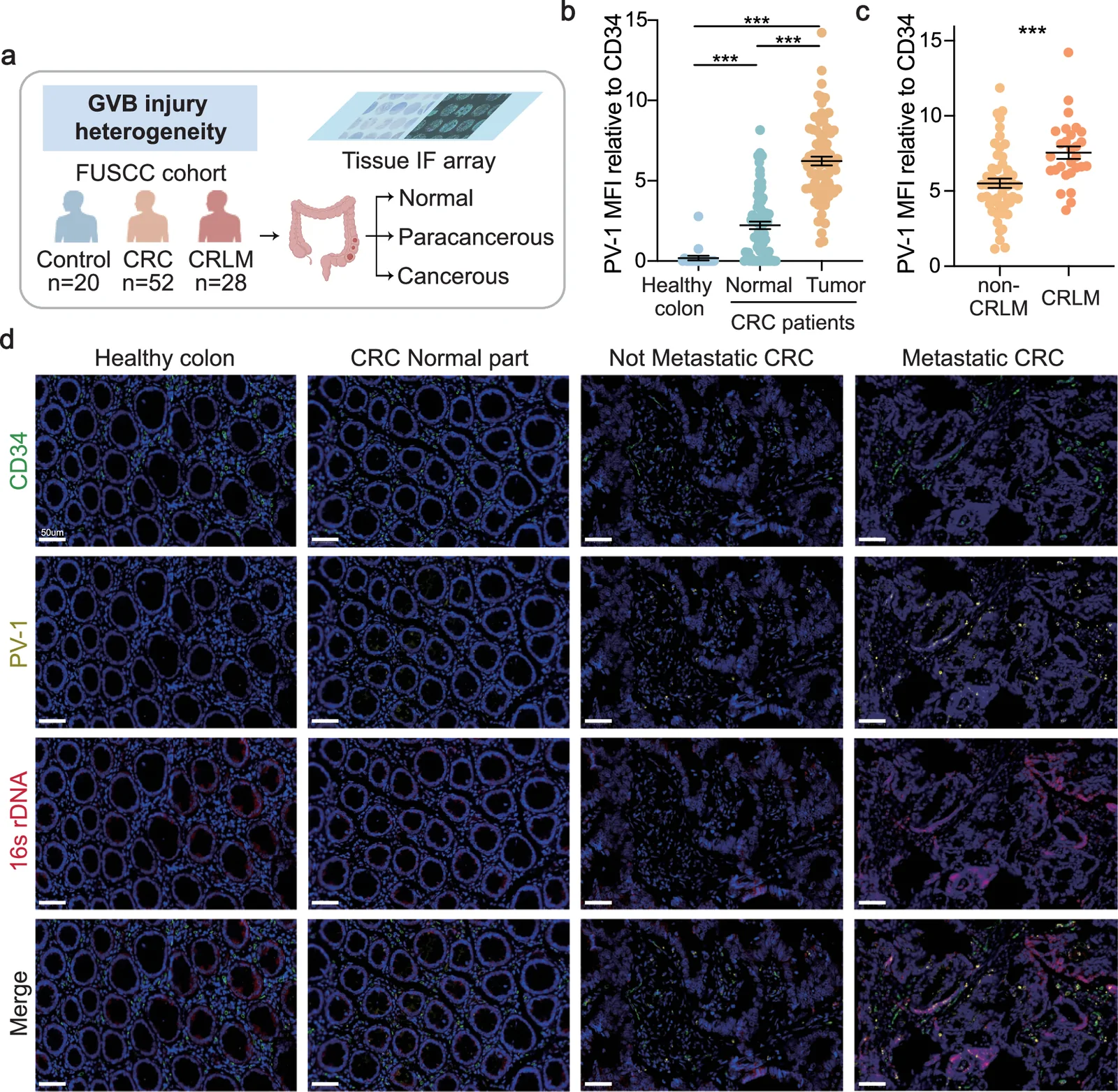
CRLM patients exhibit significant GVB impairment. **a** Schematic diagram depicting the design of the tissue immunofluorescence (IF) staining in the FUSCC cohort (healthy subjects, *n* = 20; CRC patients who developed (*n* = 28) or did not develop (*n* = 52) liver metastases) to identify heterogeneity in GVB damage. **b** Quantification of the MFI of PV-1 among the CD34-positive vessels in tumors (*n* = 80) and paired healthy mucosae (*n* = 80) from CRC patients and healthy colons (*n* = 20). **c** Quantification of the MFI of PV-1 among the CD34-positive vessels in CRC patients with (*n* = 28) or without liver metastases (*n* = 52) (excluding non-specific staining). **d** Representative images of CD34, PV-1, and 16S rDNA IF staining in FFPE samples from healthy subjects and CRC patients with/without distant metastases (magnification, 20×). Data are represented as mean ± SEM and are shown in scatter dot plots (**b**, **c**). Statistical significance was evaluated using one-way ANOVA followed by Tukey’s post hoc comparison test and a two-sided Mann‒Whitney unpaired test. ***P < 0.001.

To investigate the state of the GVB in colon vessels, we performed a retrospective analysis (with a mean follow-up of 2.1 years) of formalin-fixed paraffin-embedded (FFPE) resected human colons from CRC patients at the time of diagnosis (with or without distant metastases, *n* = 80) and from healthy subjects (*n* = 20) from the Fudan University Shanghai Cancer Center (FUSCC) training cohort (Fig. 1a; Supplementary Table S1). First, we observed that the mean fluorescence intensity (MFI) of PV-1 (relative to that of CD34) in endothelial cells was higher in CRC patients than in healthy control samples after the exclusion of non-specific staining reactions. Notably, the level of PV-1 was also higher in non-cancerous tissues from CRC patients than in tissues from healthy controls, suggesting that the GVB is altered not only inside the tumor tissue but also in the adjacent healthy mucosa (Fig. 1b, d). To assess whether higher PV-1 levels in CRC patients are correlated with distant metastases, we separated samples from CRC patients who developed (*n* = 28) and did not develop (*n* = 52) liver metastases. This analysis revealed that compared with non-metastatic patients, CRLM patients had higher PV-1 level (Fig. 1c, d), suggesting that CRLM patients may have GVB impairment. Consistent with previous findings reported by Bertocchi et al.^10^ demonstrating that *E. coli*-induced GVB impairment promotes CRC liver metastasis and that increased PV-1 expression is associated with distant recurrence, our data showed analogous observations in patients with *Eggerthella lenta* enrichment (Fig. 1a–d). These concordant findings across distinct bacterial species underscore the broader relevance of GVB disruption as a common pathogenic mechanism in CRC metastasis.

### The gut-colonizing bacterium *E. lenta* is associated with GVB damage, tumor metastasis and recurrence

**Fig. 2:**
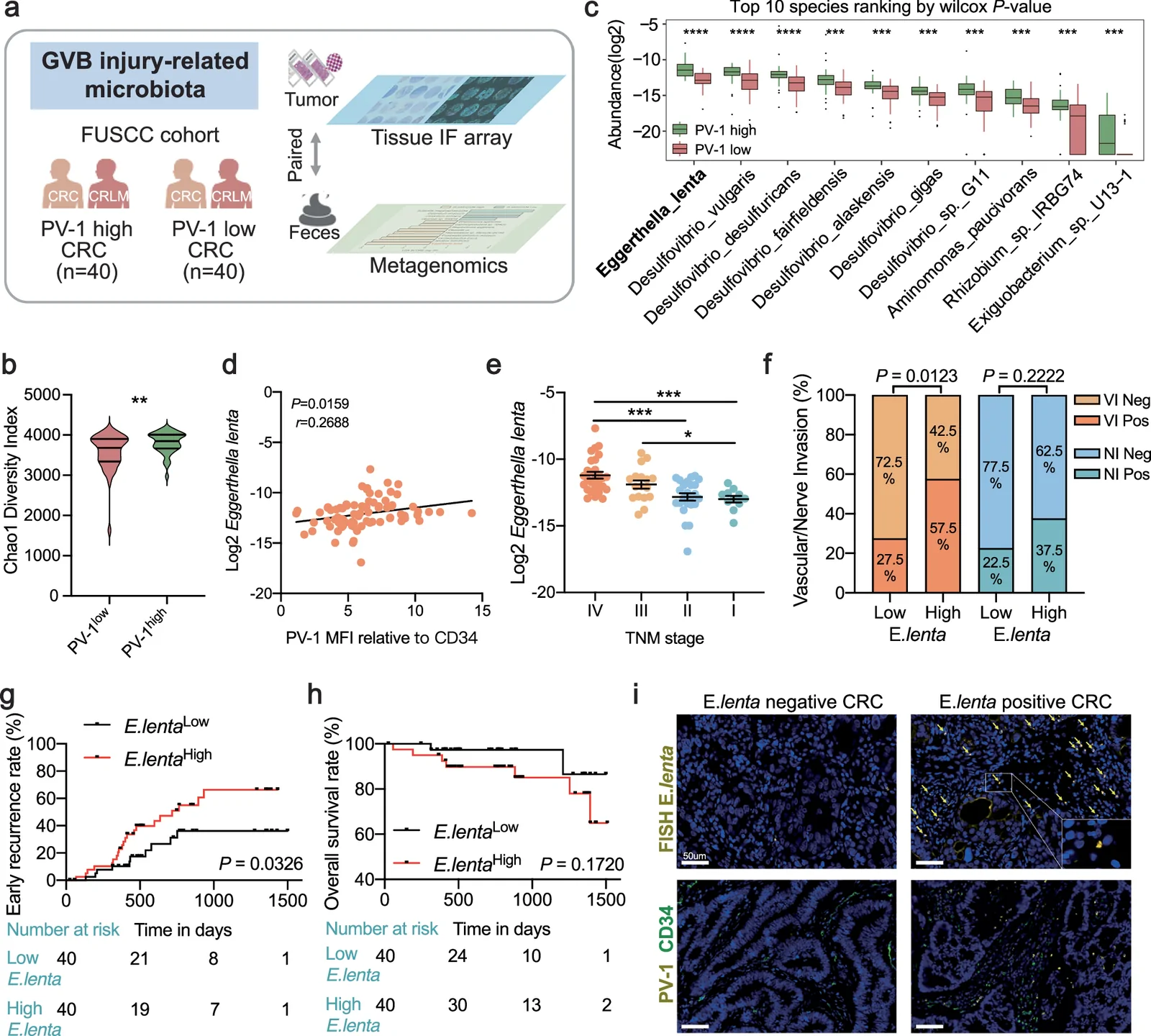
The gut-colonizing bacterium *E. lenta* is associated with GVB damage, tumor metastasis and recurrence. **a** Schematic diagram depicting the design of tissue-paired fecal metagenomics analysis on “PV-1 high” (*n* = 40) and “PV-1 low” (*n* = 40) CRC patients (according to the median level of PV-1 relative to CD34) to characterize the gut microbiota taxonomy in patients with mild and severe GVB damage. **b** Comparison of the α-diversity Chao1 index between the “PV-1 high” and “PV-1 low” groups. The diversity indices were compared using the Wilcoxon rank-sum test and controlled for the FDR (**P < 0.01). **c** Comparison of the relative abundance of the top 10 species (sorted by *P* value) between the “PV-1 high” and “PV-1 low” groups (Wilcoxon rank-sum test, ***P < 0.001). The box presents the 95% CIs; the line inside denotes the median. **d** Correlations between tumor vessel PV-1 expression and the relative abundance of fecal *E. lenta* in patients with CRC (*n* = 80; *P* < 0.05 by two-tailed nonparametric Spearman correlation; linear regression). **e** Comparison of the relative abundance of *E. lenta* in patients with different TNM stages (I–IV, one-way ANOVA followed by Tukey’s post hoc comparison test, *P < 0.05; ***P < 0.001). **f** Proportion of patients with vascular or nerve invasion (positive or negative) in “*E. lenta* high” (*n* = 40) and “*E. lenta* low” (*n* = 40) groups (according to the median relative abunda*n*ce of *E. lenta*) (Fisher’s exact test). **g**, **h** Comparison of early recurrence (**g**) and overall survival (**h**) between the “*E. lenta* high” and “*E. lenta* low” groups using Kaplan–Meier curves (log-rank (Mantel–Cox) test). **i** Representative images of *E. lenta* FISH staining and PV-1/CD34 IF staining in FFPE samples from CRC patients.

To better understand the involvement of the microbiome in GVB damage, 16S rDNA staining was performed with confocal microscopy for PV-1 and CD34. Compared with those from non-metastatic CRC patients, tumor tissues from CRLM patients were more strongly colonized by bacteria, accompanied by increased PV-1 level (Fig. 1d). Next, we stratified the patients into “PV-1 high” (*n* = 40) and “PV-1 low” (*n* = 40) groups according to the median level of PV-1 relative to CD34 and analyzed the metagenomic sequencing of paired fecal samples with the aim of characterizing the gut microbiota taxonomy in patients with mild and severe GVB damage (Fig. 2a). Univariate analyses indicated that PV-1 status was significantly associated with tumor node metastasis (TNM) stage, vascular invasion, survival, and recurrence (Supplementary Table S2). These correlations remained significant after adjustment for confounding variables (age, BMI, and sex). Compared with those in the PV-1 low group, the Chao1 richness estimates of fecal alpha diversity were significantly higher in the PV-1 high group (false discovery rate (FDR) = 0.0083, *t*-test) (Fig. 2b; Supplementary Table S3). We subsequently performed a differential abundance analysis by using Wilcoxon tests to identify the taxa present in samples with different levels of PV-1 expression. At the species level, *E. lenta* and species in the *Desulfovibrio* genus (*Desulfovibrio vulgaris*, *Desulfovibrio desulfuricans*, *Desulfovibrio fairfieldensis*, etc.) were more abundant in the PV-1 high group, indicating that these species are positively associated with an activated GVB-damaged microenvironment in CRC (Fig. 2c; Supplementary Table S4). Among these species, the relative abundance of *E. lenta* was the highest in the PV-1 high group, with the most significant difference compared with the PV-1 low group (Supplementary Tables S4 and S5). Correlation analysis also revealed that fecal *E. lenta* abundance was significantly positively correlated with tumor vessel PV-1 expression (*r* = 0.2688; *P* = 0.0159; Fig. 2d; correlations between the relative abundance of the top 10 species and that of PV-1 are shown in Supplementary Table S6). Thus, we hypothesized that *E. lenta* might be involved in GVB damage, and we conducted follow-up validation experiments. To determine whether *E. lenta* is associated with tumor progression, we compared the relative abundance of *E. lenta* between patients with different TNM stages. Unsurprisingly, *E. lenta* was significantly enriched in patients with advanced disease stages (TNM stages III/IV, especially stage IV patients with distant liver metastasis) compared with patients in early stages (TNM stages I/II) (Fig. 2e). According to the median relative abundance of *E. lenta*, we further stratified the patients into “*E. lenta* high” (*n* = 40) and “*E. lenta* low” (*n* = 40) groups (Supplementary Table S7). Notably, compared with that in the *E. lenta* low group, the proportion of patients with positive vascular invasion was significantly higher in the *E. lenta* high group (Fig. 2f). To assess the impact of *E. lenta* infection on patient clinical course, we evaluated whether increased *E. lenta* abundance was associated with early recurrence and overall survival (Fig. 2g, h). Kaplan‒Meier curves revealed that high *E. lenta* abundance was significantly associated with an increased rate of early recurrence (*P* = 0.0326). In terms of overall survival, *E. lenta* infection led to a worse prognosis, although the difference between groups was not statistically significant (*P* = 0.172). Fluorescent in situ hybridization (FISH) was used to detect *E. lenta* infection and PV-1 and CD34 expression in CRC tissues (FUSCC cohort). As shown in Fig. 2i, *E. lenta* was found to invade CRC tissues and was frequently accompanied by PV-1 overexpression. Collectively, our results highlight that the gut-colonizing bacterium *E. lenta* is associated with GVB damage, tumor metastasis and recurrence.

### Validation cohort analysis reveals the prevalence of *E. lenta* and susceptibility to organ-specific metastasis

To determine whether there is a difference in the prevalence of *E. lenta* in patients with liver metastasis and lung metastasis, we expanded our validation experiments by incorporating an independent cohort from FUSCC, comprising 30 non-metastatic CRC patients, 20 CRC patients with liver metastases, and 17 CRC patients with lung metastases (Supplementary Fig. S1a; demographic details are provided in Supplementary Table S1). Through 16S rRNA sequencing and IF co-localization analysis of primary tumor tissues, we demonstrated that compared with non-metastatic controls, both liver and lung metastasis CRC groups exhibited significantly elevated levels of *E. lenta*, vascular endothelial PV-1 expression, and ATF4 activation (corresponding to the findings in Fig. 6) (Supplementary Fig. S1b–e). Notably, no significant differences were observed between the liver and lung metastasis subgroups (*P* > 0.05 for all parameters).

Using median-based stratification, we categorized the entire cohort into high vs low *E. lenta* abundance groups (*n* = 33 vs 34) and high vs low PV-1 expression groups (*n* = 33 vs 34). *χ*^2^ analysis revealed no significant association between metastatic organotropism (liver vs lung) and either *E. lenta* abundance (*P* = 0.9421) or PV-1 expression level (*P* = 0.9689). Importantly, we identified a significant positive correlation between *E. lenta* abundance and vascular PV-1 expression across the entire validation cohort (Supplementary Fig. S1f, Pearson *r* = 0.2631, *P* = 0.0315). Furthermore, compared with patients with low *E. lenta* colonization, patients with high *E. lenta* colonization exhibited significantly elevated ATF4 expression (*P* = 0.0004 by the *χ*^2^ test).

These comprehensive validation results reinforce the robustness of our original findings while addressing potential concerns regarding predisposition to organ-specific metastasis.

### *E. lenta* destroys the tight junctions of endothelial cells and promotes the endovascular migration of CRC cells

**Fig. 3:**
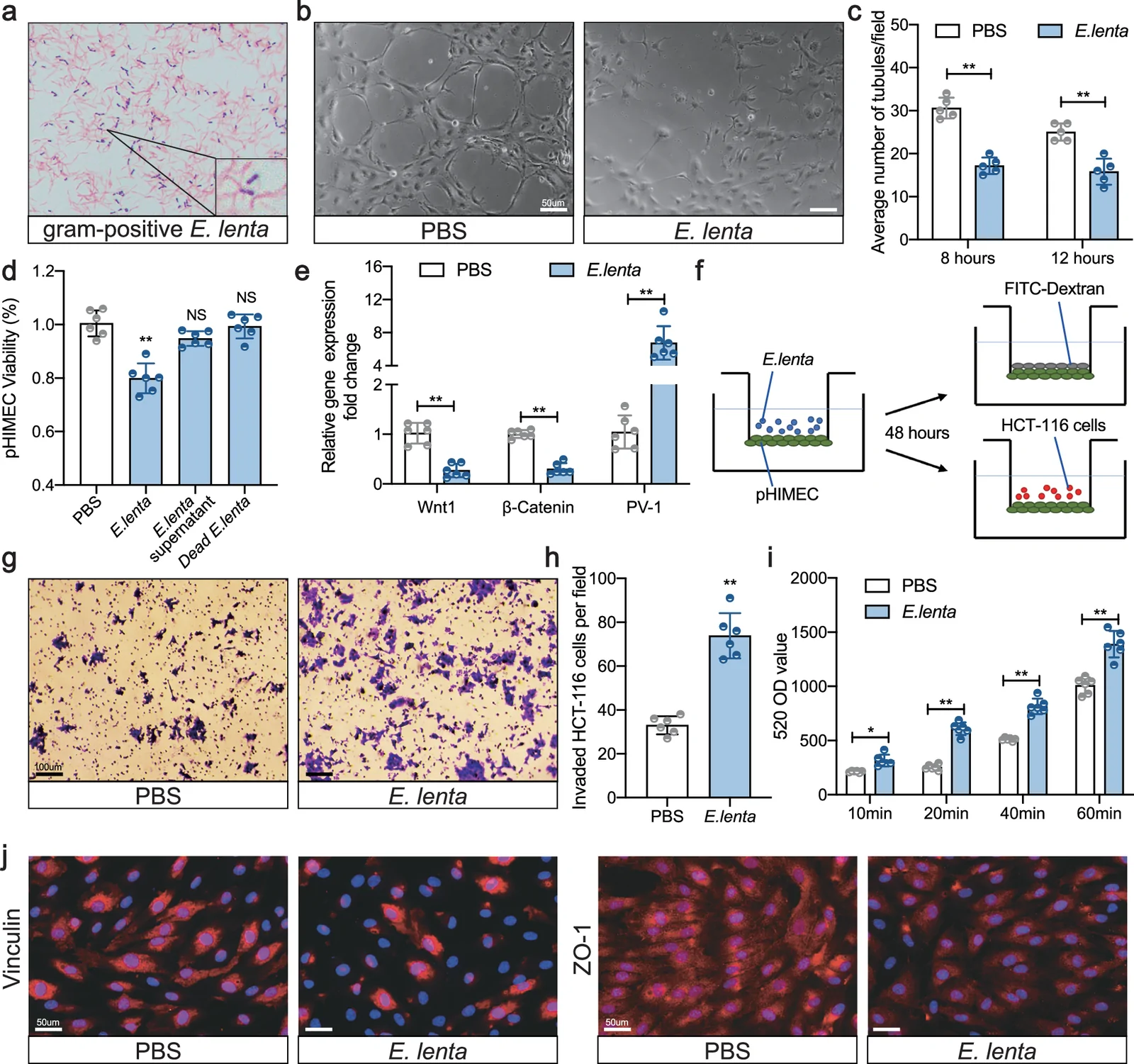
*E. lenta* destroys the tight junctions of endothelial cells and promotes the endovascular migration of CRC cells. **a** Gram staining of the anaerobic *E. lenta* standard strain (ATCC, #25559) under a microscope with immersion oil. **b** Representative bright field microscopy images of pHIMECs after 12 h co-culture with *E. lenta* (MOI of 1:1000) or PBS. **c** The average number of pHIMEC tubules per field was counted after the pHIMECs were co-cultured with *E. lenta* for 8 h or 12 h. **d** The viability of the pHIMEC was analyzed by an ATP assay after the cells were incubated with *E. lenta*, *E. lenta* supernatant, and heat-killed *E. lenta* for 12 h. **e** Quantification of Wnt1, β-catenin and PV-1 mRNA expression in pHIMECs by quantitative real-time PCR (qPCR) after the cells were co-cultured with *E. lenta* for 12 h. **f**–**i** pHIMECs were grown on the inserts of a dual-well plate for 24 h to form endothelial monolayers. For the trans-endothelial migration assay, after being co-cultured with PBS or *E. lenta* for 24 h, HCT-116 cells were loaded onto the upper chamber and allowed to migrate. The number of migrated cells in **g** was quantified (*n* = 6) (**h**). FITC-dextran was added to the top well, and the fluorescence in the bottom wells was measured at different times (**i**). **j** Representative images of IF staining for vinculin and ZO-1 in pHIMECs after 12 h of co-culture with *E. lenta* (MOI of 1:1000) or PBS. In **c**, **d**, **e**, **h**, **i**, the data are represented as mean ± SEM and are shown in scatter dot plots. Statistical significance was evaluated using a two-sided Mann‒Whitney unpaired test. *P < 0.05; **P < 0.01.

Next, we purchased an anaerobic gram-positive *E. lenta* standard strain (ATCC, #25559; Fig. 3a) to validate its involvement in controlling GVB permeability and the endovascular migration of CRC cells in vitro. When primary human intestinal microvascular endothelial cells (pHIMECs) were co-cultured with *E. lenta*, as expected, supplementation with *E. lenta* decreased the number of tubules at both 8 h and 12 h (Fig. 3b, c). We further explored whether *E. lenta* itself or its secreted products affect the viability of pHIMECs. As shown in Fig. 3d, a reduction in pHIMEC viability was observed upon exposure to live *E. lenta*, whereas treatment with heat-killed *E. lenta* and *E. lenta* supernatant had no significant inhibitory effect. With respect to bacteria‒host interactions, adhesion of the pathogen to host cells is often the first step during host regulation by living bacteria^11^. We next measured the ability of *E. lenta* and the *E. coli* DH5α control to adhere to pHIMECs in a Transwell plate under anaerobic conditions. *E. lenta* showed high adhesion capacity to pHIMECs (81.2% adherence of bacterial cells at a 1:10 dilution), while DH5α showed low adhesion capacity to these cells (only 9.7%) of the bacterial cells remained bound at a 1:10 dilution). To specifically address the pathological relevance of *E. lenta* adhesion, we systematically investigated bacterial adhesion using scanning electron microscopy (SEM). *E. lenta* was co-cultured with pHIMECs (1:10 and 1:100 dilutions; 3 independent biological replicates) for 4 h, and the samples were extensively washed with PBS (4 times, 5 min each) before imaging. SEM clearly revealed that a large amount of *E. lenta* cells adhered to the endothelial surface in a dose-dependent manner (Supplementary Fig. S2). These findings directly demonstrate that *E. lenta* robustly and stably adheres to endothelial cells under conditions that mimic the intestinal microvascular environment.

Since Wnt/β-catenin signaling is known to be associated with endothelial cell proliferation and migration^12^, the expression of Wnt1, β-catenin and PV-1 in pHIMECs was examined. *E. lenta* co-culture significantly downregulated the expression of Wnt1 and β-catenin in pHIMECs, whereas the expression of the GVB damage-related marker PV-1 increased (Fig. 3e). To assess the effect of *E. lenta* on the permeability of the endothelial monolayer, trans-endothelial invasion of cancer cells (HCT-116) was examined (Fig. 3f). Compared with pHIMECs treated with PBS, significantly more HCT-116 cells migrated through *E. lenta*-treated pHIMEC monolayers (Fig. 3g, h). Changes in the permeability of the endothelial monolayer after *E. lenta* treatment were also detected by measuring the traversal of FITC-labeled dextran through pHIMEC monolayers. Compared with cells treated with PBS, treatment of pHIMECs with *E. lenta* significantly increased the passage of the fluorescent probe through the endothelial layer (Fig. 3i). IF staining revealed marked reductions in the traversal of both vinculin and ZO-1 across pHIMEC monolayers after treatment with *E. lenta* but not after treatment with PBS (Fig. 3j). Taken together, these results suggest that *E. lenta* can adhere to and disrupt the tight junctions of endothelial cells, promoting the endovascular migration of CRC cells.

### *E. lenta* destroys the GVB and promotes CRC liver metastasis in vivo

**Fig. 4:**
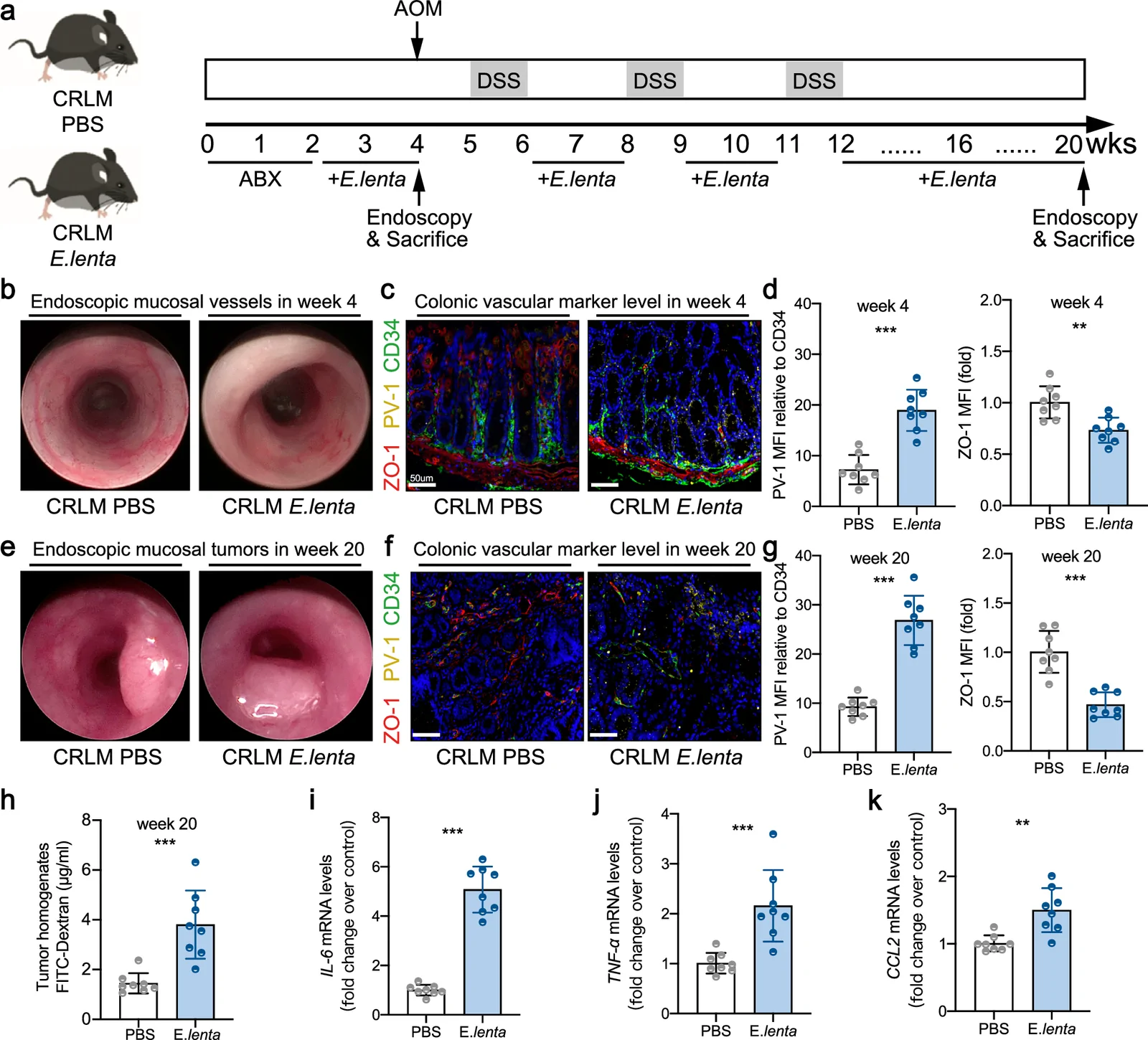
*E. lenta* destroys GVB and promotes CRC liver metastasis in vivo. **a** Scheme of the animal experiment. In brief, to demonstrate the effect of *E. lenta* on endothelial barriers in vivo, we gavaged Abx mice with live *E. lenta* or PBS for 2 weeks and then constructed AOM/DSS CRC mouse models and liver metastasis models until week 20. **b**, **e** Representative endoscopic images of mucosal vessels in Abx CRC mice administered with *E. lenta* or PBS at week 4 (**b**) and week 20 (**e**). **c**, **f** Representative images of IF staining for PV-1/CD34 and ZO-1 in the colon tissues/tumors of Abx CRC mice administered with *E. lenta* or PBS at week 4 (**c**) and week 20 (**f**). **d**, **g** Quantification of the MFI of PV-1 among the CD34-positive vessels and the MFI of ZO-1 in the colon tissues/tumors of Abx CRC mice administered with *E. lenta* or PBS at week 4 (**d**) and week 20 (**g**) (excluding non-specific staining). **h** FITC-dextran was injected into mice via the tail vein, and the leakage of FITC-dextran in tumor tissue was examined at week 20 (*n* = 8 per group). **i**–**k** Quantification of *IL-6* (**i**), *TNF-α* (**j**), and *CCL-2* (**k**) mRNA in mouse colon tumors at week 20 (*n* = 8 per group) using qPCR. In **d**, **g**–**k**, the data are represented as mean ± SEM and are shown in scatter dot plots. Statistical significance was evaluated using a two-sided Mann‒Whitney unpaired test. **P < 0.01; ***P < 0.001.

To demonstrate the effect of *E. lenta* on endothelial barriers in vivo, we gavaged mice treated with broad-spectrum antibiotics (Abx) with live *E. lenta* or PBS for 2 weeks, and AOM/DSS CRC mouse models and liver metastasis models were constructed until week 20 as described in “Materials and Methods” section (Fig. 4a). As expected, two weeks of *E. lenta* gavage before tumor model construction significantly damaged the vascular structure of the colon, increased endothelial PV-1 expression, and decreased ZO-1 expression (Fig. 4b–d). After CRC mouse model construction (week 20), we also detected increased PV-1 expression and decreased ZO-1 expression in the colon tumors of the *E. lenta* gavage group compared with those of the PBS group after the exclusion of non-specific staining reactions, and these changes occurred even before any signs of distant metastasis were detected (Fig. 4e–g). FITC-dextran was injected into the mice via the tail vein, and severe leakage of FITC-dextran into the tumor tissue was detected in the *E. lenta*-treated mice (Fig. 4h). Interestingly, although the number of intestinal tumors increased after *E. lenta* treatment, this change was not statistically significant (Supplementary Fig. S3a, b). Our mechanistic studies demonstrated that *E. lenta* primarily targets endothelial cells via endoplasmic reticulum (ER) stress pathways rather than by directly regulating tumor cell proliferation or survival. To confirm this, we conducted complementary CRC organoid co-culture experiments. *E. lenta* exposure (at a multiplicity of infection (MOI) of 1:1000 for 12 h) caused no significant changes in organoid viability (*P* = 0.89) or morphological integrity (Supplementary Fig. S3c, d), supporting our hypothesis that the pro-metastatic effects of *E. lenta* are mediated by vascular barrier disruption rather than direct tumor cell modulation. We then evaluated whether the levels of inflammatory cytokines and chemokines were also altered. Our results revealed that *E. lenta* treatment caused significant increases in *IL-6*, *TNF-α*, and *CCL-2* gene expression in the colon tumors of mice (Fig. 4i–k). To further determine whether *E. lenta* treatment can promote tumor metastasis, we expanded our experimental system using orthotopic CRLM models (Fig. 7a). The results revealed that *E. lenta* infection significantly increased the liver metastatic burden by increasing both the proportion of mice that developed liver metastases and the total surface area of the metastatic sites (Fig. 7b–e). Taken together, our results suggest that *E. lenta* can destroy the GVB and promote CRC liver metastasis in vivo.

### *E. lenta* may regulate ER stress and autophagy pathways in tumor endothelial cells

**Fig. 5:**
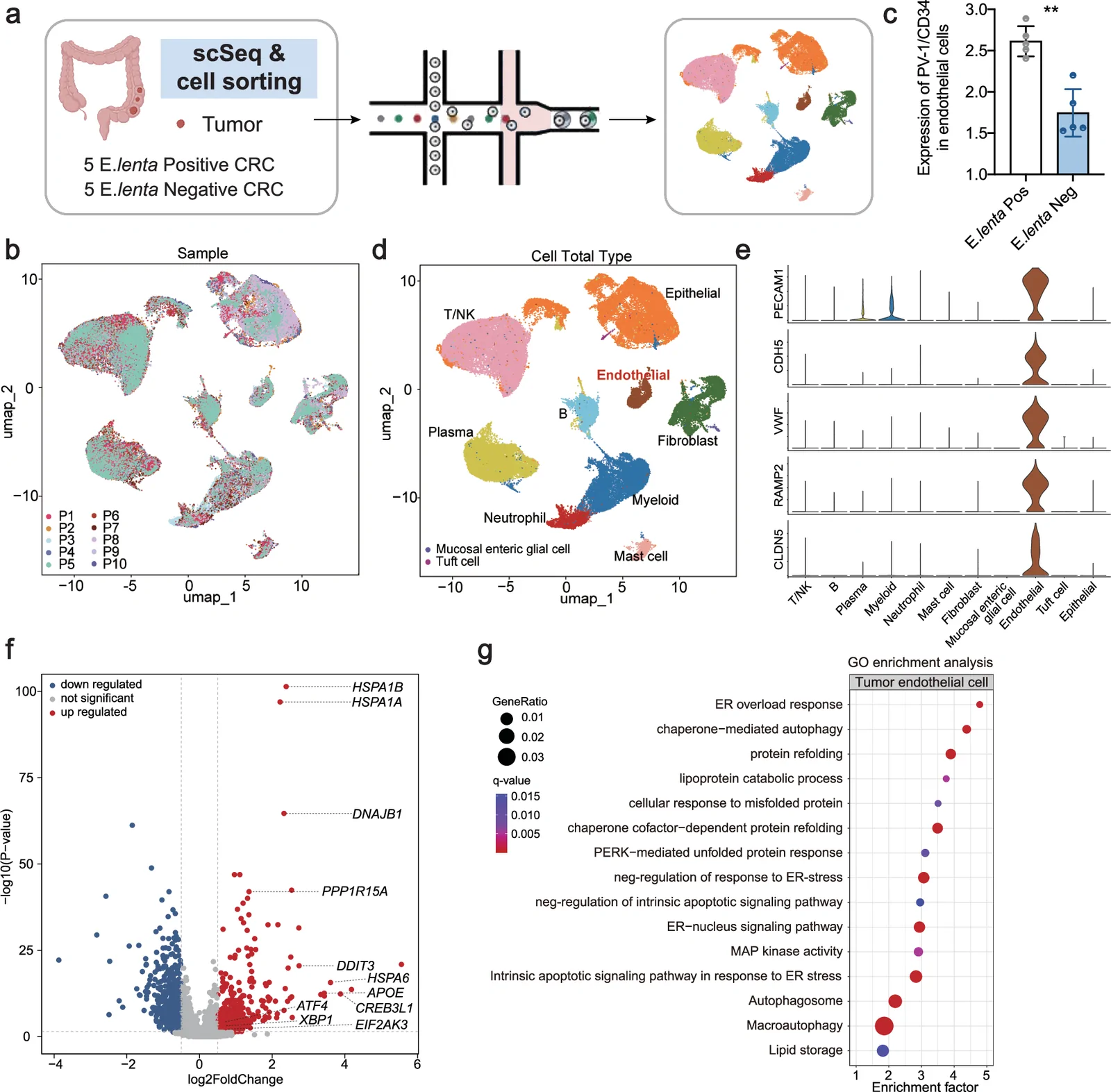
*E. lenta* may regulate ER stress and autophagy pathways in tumor endothelial cells. **a** Schematic diagram depicting the design of the scRNA-seq analysis of five *E. lenta*-positive CRC tissues and five *E. lenta*-negative CRC tissues to explore the regulatory mechanism underlying the effect of *E. lenta* on tumor endothelial cells and the GVB. **b** Uniform manifold approximation and projection (UMAP) plots for 97,257 high-quality cells showing the sample origin. **c** Quantification of PV-1 expression among the CD34-positive vessels in *E. lenta*-positive and *E. lenta*-negative CRC tissues (*n* = 5 per group; Wilcoxon rank-sum test, **P < 0.01). **d** UMAP plots showing the cell types for the 97,257 cells. **e** Violin plots showing the expression distribution of endothelial marker genes in 11 cell types. **f** Volcano plot for DEGs in endothelial cell clusters comparing the *E. lenta*-negative group to the *E. lenta*-positive group (using a cutoff of abs(avg_log_2_FC) > 0.5 and a *P* value < 0.05). Blue represents downregulated genes, red represents upregulated genes, and gray represents non-significant genes. **g** GO analyses of significantly differentially expressed genes in endothelial cell clusters (FDR value < 0.05). The gene ratio corresponds to the percentage of genes enriched in a GO term.

To explore the regulatory mechanisms underlying the effects of *E. lenta* on tumor endothelial cells and the GVB, single-cell RNA sequencing (scRNA-seq) and cell sorting were performed on five *E. lenta*-positive CRC tissue samples and five *E. lenta*-negative CRC tissue samples (Fig. 5a, b). Unsurprisingly, PV-1 levels in tumor vessels were significantly higher in *E. lenta*-positive patients than in *E. lenta*-negative patients (Fig. 5c). After the removal of low-quality cells (see “Materials and Methods” section), 97,257 cells were retained for biological analysis. After normalization of gene expression and principal component analysis, we used graph-based clustering to partition the cells, which could be further assigned to 11 known cell lineages through marker genes (Fig. 5d, e): epithelial cells (marked by *EPCAM*), endothelial cells (marked by *PECAM1*, *CDH5*, *VWF*, *RAMP2*, and *CLDN5*), T/NK cells (marked by *CD3D* and *NKG7*), B cells (marked by *MS4A1* and *CD79A*), plasma cells (marked by *MZB1*), fibroblasts (marked by *COL3A1*), neutrophils (marked by *FCGR3B*), myeloid cells (marked by *LYZ*), mast cells (marked by *TPSB2*), tuft cells (marked by *SH2D6*), and mucosal enteric glial cells (marked by *PLP1*).

Gene Ontology (GO) analysis of total cells revealed that the *E. lenta*-positive group exhibited significant enrichment of endothelial cell-specific pathways, including “endothelial cell activation” (Enrichment Factor (EF) = 5.0306, FDR = 0.0119), “regulation of endothelial cell development” (EF = 3.9061, FDR = 0.0171), and “positive regulation of endothelial cell apoptotic process” (EF = 3.228, FDR = 0.0239) (Supplementary Fig. S4a, b and Table S11). In other cell types, although we observed a reduction in the proportion of epithelial cells in *E. lenta*-positive samples, this likely reflects secondary effects of intestinal barrier and endothelial disruption. Next, we focused on differences in gene expression in endothelial cells between the two groups of patients. With a cutoff of abs(avg_log_2_fold change (FC) > 0.5 and a *P* value < 0.05, 216 genes were determined to be differentially expressed genes (DEGs) in the *E. lenta*-positive CRC group compared with the control group (Fig. 5f; Supplementary Table S8). The GO biological processes associated with the upregulated genes in tumor endothelial cells from the *E. lenta*-positive CRC group were largely related to ER stress and autophagy (Fig. 5g; Supplementary Table S9). Collectively, the results of scRNA-seq revealed that *E. lenta* may regulate ER stress and autophagy pathways in tumor endothelial cells.

### *E. lenta* surface proteins induce ER stress in pHIMECs via a TLR4-dependent signaling cascade

To identify the pathogenic component(s) that mediate ER stress induction, we performed treatment experiments using washed *E. lenta* or *E. lenta* culture supernatants. As shown in Supplementary Fig. S5a, *E. lenta* itself induced ER stress responses (as evidenced by p-PERK upregulation) and autophagic activation (Beclin-1 increase) in pHIMEC cells, whereas culture supernatant alone had minimal effects. Notably, SEM (Supplementary Fig. S2) confirmed the direct physical interaction between *E. lenta* and vascular endothelial cells. These findings suggest that surface-associated components rather than secreted metabolites mediate the observed effect.

Given the established role of TLR4 in bacterial recognition, we investigated its involvement in this process using the specific inhibitor TAK-242. As shown in Supplementary Fig. S5b, TAK-242 pretreatment reversed *E. lenta*-induced changes in the expression of ER stress markers (p-PERK and p-eIF2α) and autophagic activation in pHIMECs. Importantly, neither DH5α control bacteria nor TAK-242 alone induced significant ER stress responses. This selective inhibition confirms that TLR4 is a key receptor that mediates the effects of *E. lenta* on endothelial stress responses.

To further validate these mechanistic findings in a clinically relevant context, we examined ER stress markers in human CRC tissues stratified by *E. lenta* colonization status. Immunohistochemical staining revealed that compared with *E. lenta*‑negative tissues, *E. lenta*‑positive CRC tissues exhibited significantly higher expression of both BiP and p‑eIF2α (Supplementary Fig. S6). These results provide direct in vivo evidence that *E. lenta* colonization is associated with elevated ER stress in the tumor microenvironment, strongly corroborating our in vitro observations and reinforcing the translational relevance of the TLR4‑ER stress axis in *E. lenta*‑associated CRC metastasis.

### Knockdown of PERK protects against GVB damage and tumor metastasis induced by the *E. lenta*-ER stress pathway

**Fig. 6:**
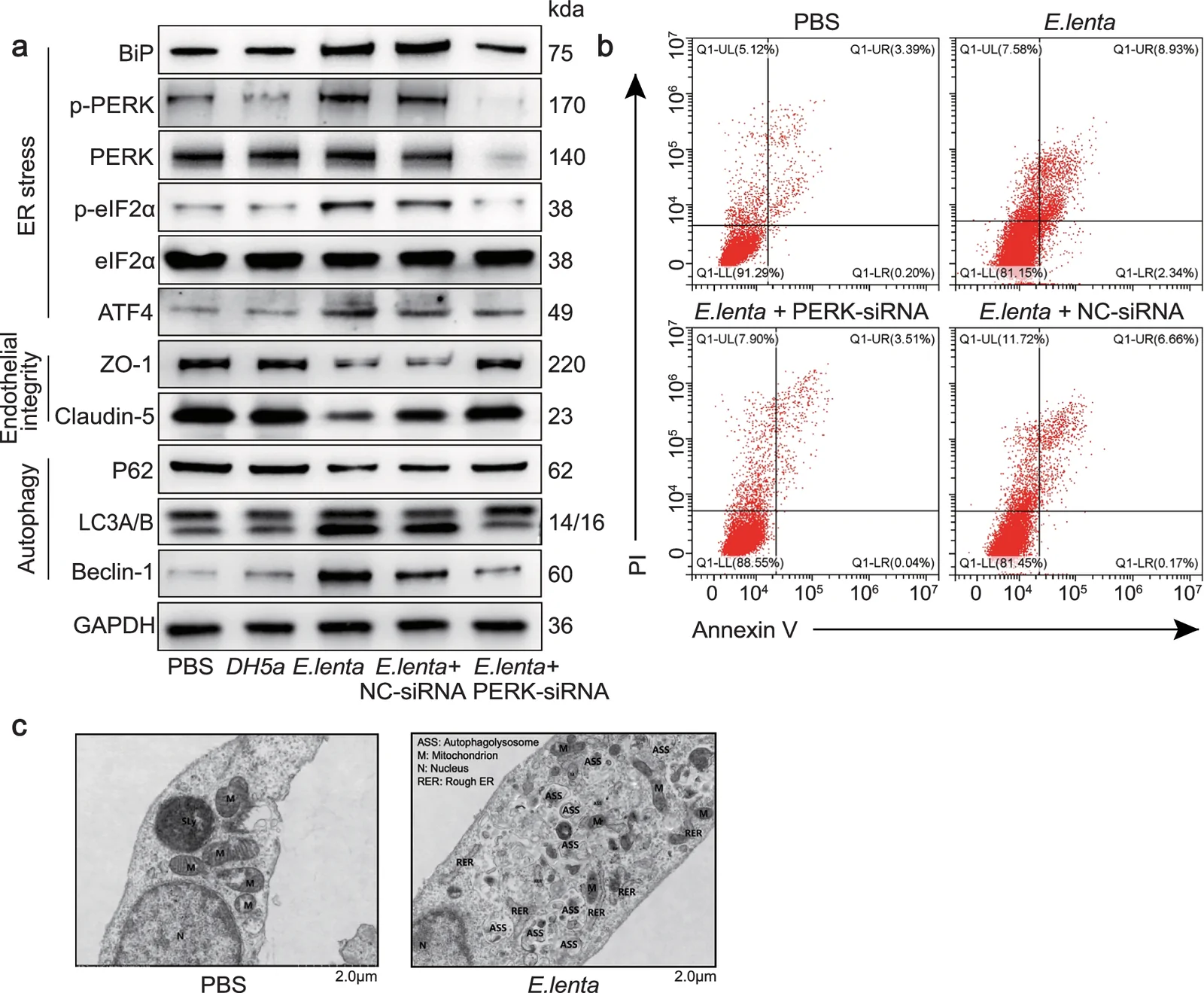
Knockdown of PERK protects against GVB damage and tumor metastasis induced by the *E. lenta*-ER stress pathway. **a** pHIMECs were treated with PBS, *E. coli* DH5α, or *E. lenta* along with PERK-siRNA and then analyzed by western blot for the protein levels of ER stress, endothelial integrity, and autophagy markers. **b** Annexin V/PI staining of pHIMECs after treatment with *E. lenta* along with PERK-siRNA was analyzed with an Annexin V-PI apoptosis detection kit by flow cytometry. **c** After being treated with either PBS or *E. lenta*, the pHIMECs were visualized by transmission electron microscopy. N nucleus, RER rough endoplasmic reticulum, M mitochondrion, ASS autolysosome, SLy secondary lysosome.

On the basis of the above findings from omics analysis, we analyzed the protein expression of ER stress- and autophagy-related genes in pHIMECs. Western blot analysis revealed that compared with *E. coli* DH5α or PBS treatment, *E. lenta* treatment upregulated BiP, p-PERK, p-eIF2α, ATF4, LC3A/B and Beclin-1 expression and downregulated P62, ZO-1 and Claudin-5 expression in pHIMECs (Fig. 6a). In addition, *E. lenta* treatment significantly induced apoptosis in pHIMECs (Fig. 6b). Electron microscopy revealed the formation of autophagosomes in pHIMECs after treatment with *E. lenta* (Fig. 6c). These data indicated that ER stress and autophagy/apoptosis were induced after the pHIMECs were treated with *E. lenta*. ER stress is known to increase the kinase activity of p-PERK to promote eIF2α phosphorylation, resulting in the downregulation of protein synthesis and cell growth^13^. We further used RNA interference to knock down PERK in pHIMECs and reassessed the effect of *E. lenta* on PERK-downregulated pHIMECs. The results demonstrated that PERK siRNA reversed *E. lenta*-induced ER stress and autophagy/apoptosis, and the protein expression of BiP, p-eIF2α, ATF4, LC3A/B, Beclin-1, ZO-1, and Claudin-5 was also restored (Fig. 6a, b), indicating that ER stress was responsible for the downregulation of autophagy- and tight junction-related proteins in pHIMECs.

**Fig. 7:**
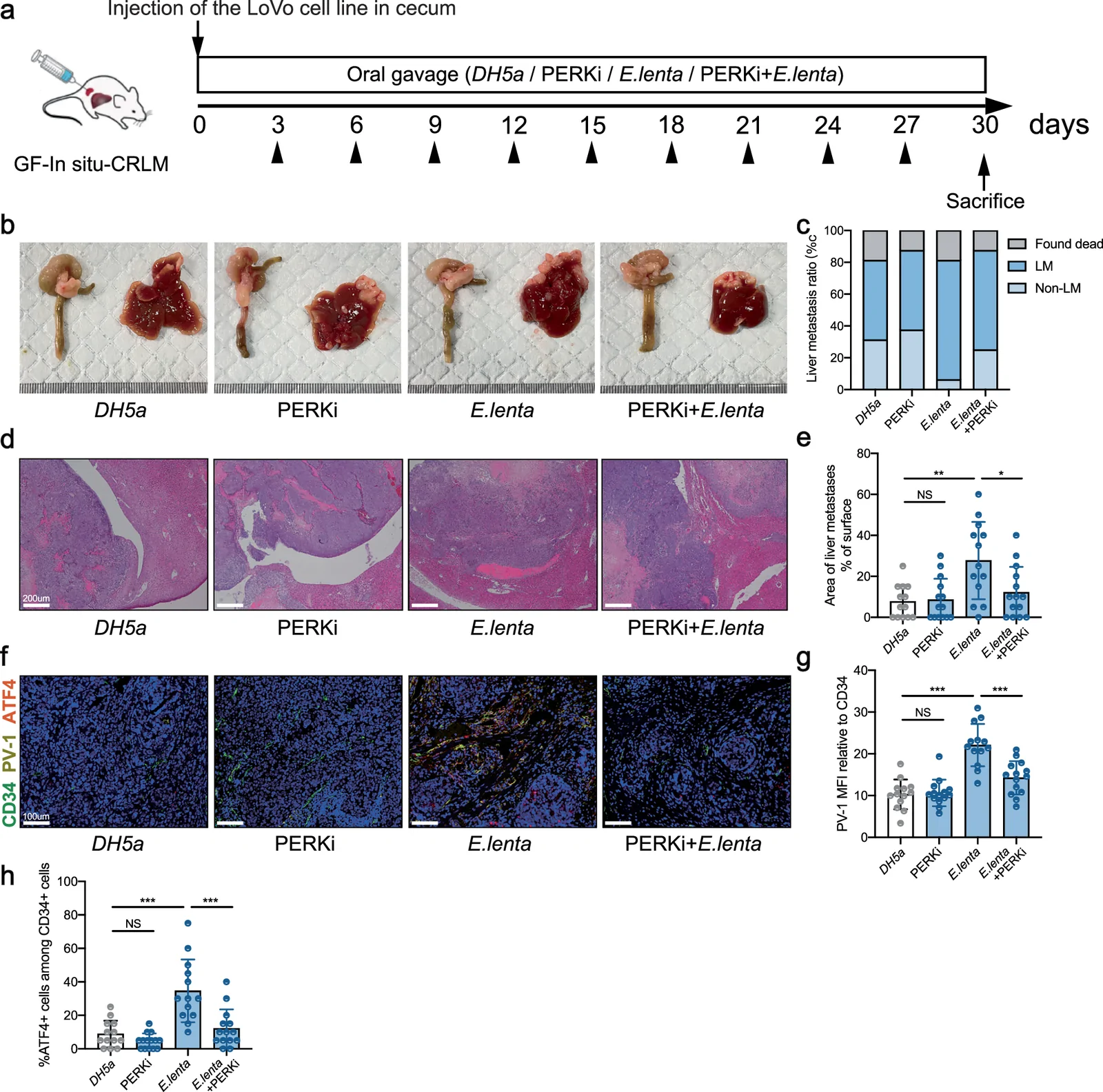
Germ-free orthotopic CRLM models reveal that GSK2606414 inhibits the *E. lenta*-driven p-PERK activation axis. **a** Scheme of the animal experiment. In brief, we established germ-free nude mice via injections of LoVo cells into the mesenteric triangle of the cecum to simulate primary colon cancer. The mice were given the PERK inhibitor GSK2606414 and/or *E. lenta* by enema every 3 days for 30 consecutive days. **b**, **d** Representative images of harvested orthotopic colon tumors, liver metastases (**b**), and liver hematoxylin and eosin (H&E) staining (**d**) in mice. **c** Liver metastasis rate and mortality in each group of mice. **e** Percentages of liver surfaces covered with macrometastatic nodules (*n* = 13–14 per group). **f** Representative images of IF staining for PV-1/CD34 and ATF4 in orthotopic colon tumors from mice administered with *E. lenta* and/or GSK2606414 for 30 days. **g**, **h** Quantification of the MFI of PV-1 among the CD34-positive vessels and the MFI of ATF4 in mice (excluding non-specific staining). In **e**, **g**, **h**, the data are represented as mean ± SD and shown in scatter dot plots. Statistical significance was evaluated using one-way ANOVA followed by Tukey’s post hoc comparison test. *P < 0.05; **P < 0.01; ***P < 0.001.

To rigorously validate the functional role of PERK signaling in *E. lenta*-induced metastasis, we comprehensively expanded our experimental system using orthotopic CRLM models (Fig. 7a). Our results revealed that *E. lenta* infection significantly increased the liver metastatic burden (Fig. 7b–e), upregulated the expression of PV-1 in vascular endothelial cells, and was accompanied by activation of the ER stress marker ATF4 (Fig. 7f–h). Co-treatment with GSK2606414 reversed these effects. In addition, monotherapy with GSK2606414 did not significantly affect metastasis, GVB integrity, or ER stress markers.

These data provide causal evidence that PERK signaling acts as a critical downstream effector of *E. lenta*-induced ER stress to promote metastatic progression. In terms of therapeutic efficacy, GSK2606414 specifically targets the bacterial infection-PERK axis, highlighting its potential for clinical translation to prevent metastasis in CRC patients with gut dysbiosis.

## Discussion

The GVB is an important anatomical structure for maintaining homeostasis and protective compartmentalization in healthy individuals. Disruption of GVB integrity may contribute to ectopic colonization by gut bacteria or liver metastasis of CRC^2^. Previous studies have confirmed that gut bacteria are among the key factors that cause GVB disruption, which is a prerequisite for non-alcoholic steatohepatitis development or CRC liver metastasis^14^. Some studies have also demonstrated that specific pathogens, such as *S. typhimurium*, *E. coli* C17, and *Fusobacterium nucleatum*, can cause GVB disruption, whereas *Akkermansia muciniphila* may restore the GVB in vivo and in vitro^2,15,16^. However, to our knowledge, no studies have characterized the gut microbiome or analyzed key bacteria in patients with CRLM or severe GVB disruption. Here, we showed that patients with high levels of PV-1, which indicated severe GVB disruption, had higher abundance of *E. lenta* and species in the *Desulfovibrio* genus than patients with low levels of PV-1. Notably, the top bacteria with differential abundance in patients with different PV-1 levels did not include bacteria that have been reported to cause GVB disruption in animal models. Therefore, we hypothesized that other key pathogens may be responsible for GVB disruption in patients with CRLM. Our FISH assay of tumor tissues derived from patients with CRLM confirmed the enrichment of *E. lenta* at sites of GVB disruption. On the basis of these findings, we performed further analyses and found that a high abundance of *E. lenta* was associated with more advanced disease stages, more significant vascular or nerve invasion, and an increased rate of early recurrence, suggesting a potential role for *E. lenta* in CRC metastasis.

*E. lenta* is an anaerobic, gram-positive bacillus that commonly colonizes the human digestive tract. However, much clinical evidence suggests that *E. lenta* can break through the gut barrier to cause severe bacteremia as well as inflammation of extra-intestinal organs in disease states^17–19^. Interestingly, a case report from 2010 claimed a polymicrobial bloodstream infection caused by *E. lenta* and *D. desulfuricans*, both of which were among the top species enriched in the PV-1 high group in our study^20^. Our in vitro experiments revealed that *E. lenta* intervention disrupted the viability of human intestinal microvascular endothelial cells (HIMECs) as well as intercellular tight junctions and consequently increased the permeability of the endothelial monolayer. Our in vivo experiments demonstrated that the disruption of the GVB by *E. lenta* provides a critical pre-metastatic niche for CRC cells and significantly promotes liver metastasis of CRC in germ-free mice. Consistent with our results, Bai et al. reported enrichment of *E. lenta* in cigarette-exposed mice with an impaired gut barrier, suggesting that the effects of *E. lenta* on gut barrier disruption may occur in a variety of pathophysiological processes^21^.

Mechanistically, we showed that the PV-1 expression level of endothelial cells in tumor samples from *E. lenta*-positive patients was much higher than that in *E. lenta*-negative patients. Hyperactivation of the ER overload response and overexpression of vascular impairment markers were observed in tumor endothelial cells from the *E. lenta*-positive group. We confirmed activation of ER stress and autophagy pathways as well as decrease in adhesion-associated proteins in HIMECs after *E. lenta* intervention, and these effects could be reversed by RNA interference of PERK. According to previous studies, the abundance of *E. lenta* is strongly associated with inflammation in and around the gut. Alexander et al. reported that *E. lenta* can induce intestinal Th17 activation in a Rorc-dependent manner and can worsen colitis in mice^22^. Balakrishnan et al. reported that *E. lenta* can increase the numbers of CXCL5- and CD4-positive T cells and both interleukin-17- and interferon-γ-producing B cells to cause systemic senescence-like inflammation^23^. Here, we report a novel form of *E. lenta*-induced vascular endothelial cell dysfunction mediated by direct contact with bacteria that can be restricted by inhibiting PERK activation. Notably, in addition to *E. lenta*, other gut bacteria have been implicated in CRC metastasis. For instance, *E. coli* C17 disrupts the GVB and modulates WNT/β‑catenin signaling^10^, whereas *F. nucleatum* activates this pathway via FadA. The absence of *E. coli* enrichment in our primary tumor cohort does not contradict prior findings. As demonstrated by Bertocchi et al., *E. coli* C17 can translocate from the gut to the liver to establish a premetastatic niche without necessarily persisting at detectable levels in the primary tumor at resection. Thus, our data do not exclude a role for *E. coli* in CRC metastasis; rather, they suggest that multiple distinct bacterial species, including *E. lenta*, can promote liver metastasis through convergent or complementary mechanisms. In contrast to the WNT-dependent pathways described for other bacteria, our findings reveal that *E. lenta* disrupts the GVB and promotes CRC liver metastasis via a distinct TLR4-dependent ER stress/autophagy pathway, without direct involvement of canonical WNT signaling.

While *E. lenta* did not directly promote CRC cell proliferation, we cannot completely exclude a direct effect on tumor cell migration. Nevertheless, our data demonstrate that *E. lenta* disrupts the GVB and that this disruption is sufficient to enhance CRC cell transendothelial migration. Moreover, in our intravenous metastasis model, *E. lenta* colonization promoted liver metastasis in the absence of direct bacterium–tumor cell contact. Therefore, we propose that GVB disruption is a primary mechanism underlying *E. lenta*‑associated metastasis. Future studies should directly examine whether *E. lenta* or its metabolites can enhance CRC cell migration independently of endothelial cells.

Our in vitro data revealed that *E. lenta* directly downregulates the expression of tight junction proteins via the PERK/ER stress pathway. However, we cannot exclude the possibility that endothelial cell death (occurring as early as 8–12 h) also contributes to increased permeability. The loss of individual endothelial cells creates physical gaps that may facilitate tumor cell passage independent of tight junction integrity. Furthermore, *E. lenta* may compromise the intestinal epithelial barrier, as suggested by our scRNA‑seq data showing reduced epithelial cell abundance and downregulation of epithelial junctional genes. Thus, the net effect on the GVB in vivo likely reflects a combination of direct endothelial junctional disruption, endothelial cell death, and potential indirect effects from epithelial injury. Future studies using genetic or pharmacological tools to uncouple cell death from junctional loss are needed to fully elucidate the individual contributions of these factors.

In conclusion, for the first time, this study demonstrates that *E. lenta* promotes CRC liver metastasis by disrupting GVB. *E. lenta* induces ER stress and autophagy in vascular endothelial cells, increases GVB permeability, and provides a pre-metastatic niche for CRC cells. *E. lenta* blooms are among the hallmarks of gut dysbiosis in patients with CRLM. Therefore, targeting *E. lenta* or the ER stress pathway in GVB may be a potential strategy to prevent CRC liver metastasis.

### Limitations of the study

While our current study established the ER stress pathway as a critical mediator of *E. lenta*-induced GVB disruption, future investigations employing targeted proteomic analyses (e.g., lipoprotein profiling) and functional mutagenesis approaches will be essential for identifying the specific bacterial components responsible for this pathogenic mechanism. The identification of specific virulence factors could unveil novel therapeutic targets for preventing bacterial-driven metastasis in CRC patients. Antibiotic pretreatment or germ‑free conditions may compromise baseline barrier function and reduce microbial competition, potentially sensitizing the gut to *E. lenta*. Nevertheless, the consistent in vitro data from this study support a direct pathogenic effect. The relatively short follow‑up (mean of 2.1 years) precludes conclusions about long‑term survival or late metastasis; extended follow‑up is ongoing and will be reported separately. While a deeper investigation of endothelial–immune crosstalk using our scRNA‑seq data could be informative, the current study focused on the direct endothelial response. Future studies with dedicated immune profiling will address whether *E. lenta* indirectly remodels the microenvironment through immune cells. Additionally, although our scRNA‑seq data revealed enrichment of ER stress‑related transcripts in endothelial cells from *E. lenta*‑high patients and in vitro experiments confirmed PERK pathway activation and functional relevance, we were unable to perform immunohistochemical validation of ER stress markers in patient tissues because of limited sample availability and technical challenges associated with p‑PERK staining in FFPE sections. Future prospective cohorts with dedicated tissue collection will be needed to confirm the spatial localization of ER stress activation in situ. Finally, the specific *E. lenta* surface ligand that engages TLR4 remains to be identified, but the genetic intractability of this bacterium precluded such an analysis in this study.

## Materials and methods

### Patient and public involvement

Patients and/or the public were not involved in the design, conduct, reporting, or dissemination of this research.

### Patient consent for publication

Written informed consent was provided by all subjects before sampling.

### Characteristics of the participants and sample collection

FFPE colonic tissue samples and paired fecal samples from patients with sporadic CRC and healthy controls were retrieved from the Fudan University Shanghai Cancer Center, Shanghai, China (FUSCC cohort). After inclusion and exclusion screening, a total of 100 eligible subjects were included in our study, including 20 healthy controls, 52 pre-operative CRC patients without distant metastases, and 28 pre-operative patients with CRLM. An independent cohort from FUSCC was enrolled, comprising 30 non-metastatic CRC patients, 20 CRLM patients, and 17 CRC patients with lung metastases. Fresh tumor tissues from an additional 10 CRC patients were collected during surgery (scRNA-seq cohort).

FFPE normal, para-cancerous, and cancerous tissues were used for IF staining. Fecal samples were collected preoperatively in sterile tubes and then stored at –80 °C prior to metagenomics screening. Fresh tumor tissue samples were obtained for scRNA-seq. None of the participants were treated with antibiotics or probiotics for one month before enrollment in this study.

Patients with sporadic CRC were excluded on the basis of the following criteria: a history of familial CRC, a history of inflammation-associated CRC, a history of irritable bowel syndrome, the presence of other coexisting malignant tumors, and a history of stool sampling not before colonoscopy or neoadjuvant therapy before stool sampling. The recruited CRC patients were divided into a CRC group (without distant metastases) and a CRLM group on the basis of the presence of liver metastases, as well as the PV-1 high group and PV-1 low group according to the median expression of PV-1 relative to that of CD34. For the healthy control group, volunteers who were confirmed with no gastrointestinal tumors after colonoscopy screening were recruited. The clinical pathological features of the CRC patients included age, sex, body mass index, TNM stage, tumor size, tumor differentiation, vascular invasion, neural invasion, survival, and tumor recurrence. The clinical information of the patients is provided in Supplementary Table S1.

### Mouse models

Experimental protocols were approved by the Institutional Animal Care and Use Committee of Fudan University Shanghai Cancer Center, and mouse experiments were conducted in accordance with the National Institutes of Health Guidelines for the Care and Use of Laboratory Animals (No. FUSCC-IACUC-S2023-0002). C57BL/6J specific-pathogen-free (SPF) mice were purchased from Shanghai SLAC Laboratory Animal Co., Ltd. All the mice were housed in an SPF facility at Shanghai SLAC Laboratory Animal Co., Ltd., with ad libitum access to chow and water.

#### Protocol 1

Eight-week-old C57BL/6J male mice were randomized into two groups: the CRLM control group and the CRLM *E. lenta* group. After they had adapted for one week, the mice received an antibiotic cocktail (Abx) via the drinking water (0.2 g/L ampicillin, neomycin, and metronidazole, and 0.1 g/L vancomycin) for two weeks to deplete the gut microbiota. Afterward, 10^9^ colony-forming units (per mouse) of live *E. lenta* resuspended in PBS or PBS was administered to the mice by gavage for 14 consecutive days. After gavage, DNA from the fecal samples (0.1 g per mouse) was extracted using a PowerFecal Pro DNA Kit (QIAamp). Total gene copies of DNA were subsequently assessed by qPCR for each fecal sample to determine the relative *E. lenta* abundance. At week 4 after gavage, some mice were sacrificed (eight animals/group) to harvest serum and colon tissue for analysis.

After the above gavage treatment was completed, another eight mice in each group were intraperitoneally injected with 10 mg/kg body weight azoxymethane (AOM; Sigma, USA). After 7 days, AOM-injected mice received dextran sulfate sodium (DSS) (1.5% w/v) in the drinking water for 7 successive days. After DSS treatment, the mice were allowed to recover for 14 days. This schedule was repeated for 3 cycles. During each recovery interval, the mice were given live *E. lenta* strains or PBS by gavage.

#### Protocol 2

Orthotopic CRLM models: We established germ-free nude mice with injections of LoVo cells into the mesenteric triangle of the cecum to simulate primary colon cancer. The mice were randomly assigned to four groups (*n* = 16/group): the *E. coli* DH5α control group; the group treated with the PERK inhibitor GSK2606414 (30 mg/kg); the *E. lenta* group; and the *E. lenta* + GSK2606414 co-treatment group. All interventions were delivered by gavage every 3 days for 30 consecutive days, followed by systematic collection of primary tumors, serum samples, and liver tissues.

### Tumor vascular FITC-dextran permeability assay

FITC-dextran (50 mg/kg; 70 kDa; Sigma) was injected into the mice via the tail vein. Tumor tissue homogenates were collected 4 h later, and the concentration of FITC-dextran in tumor samples was determined by fluorescence intensity (CLARIOstar Plus Microplate Reader; BMG Labtech, Germany).

### Culture conditions

*E. lenta* (ATCC, #25559), the typed strain of the species *E. lenta*, was obtained from American Type Culture Collection (ATCC, USA) and was cultured in tryptic soy broth (TSB, Corning, USA) medium following a previously described protocol^23^. The quantity of the bacterium was measured using serial dilution and plating on TSB agar plates. The purity of the bacterium was verified by qPCR with specific primers (F: 5′-ATACTAGGTGTGGGGGGCTCCG-3′ and R: 5′-TTTCCCCGGCTTCACGTCCATG-3′). *E. coli* strain DH5a (Invitrogen, CA, USA) was propagated aerobically in Luria–Bertani (BD Biosciences, Difco, MD, USA) medium at 37 °C.

pHIMECs were provided by BeiNa Culture Collection (BNCC379539; Beijing, China). The pHIMECs were cultured in endothelial cell medium (ScienCell, CA, USA), and the culture vessel was pre-coated with fibronectin (5 μg/cm^2^; Roche, Mannheim, Germany). The human CRC cell line HCT-116 was obtained from the ATCC. All the cell lines were verified by short tandem repeat analysis and cultured under appropriate conditions. The cells were also tested for mycoplasma using the MycoAlert Mycoplasma Detection Kit (Lonza, Belgium). All experiments were performed with cell lines cultivated for less than 12 passages after their procurement.

### Endothelial permeability and trans-endothelial migration analysis

For the endothelial permeability analysis, pHIMECs (2 × 10^4^) were seeded on polyethylene terephthalate Transwell filters in a 24-well plate (0.4 μm pore size; BD Biosciences; USA) and allowed to reach confluence, followed by addition of PBS or *E. lenta* (MOI of 1:1000), and continued culturing for 24 h. FITC-dextran (Sigma, USA) was added to the top well to reach a final concentration of 10 mg/mL. The appearance of fluorescence in the bottom well, which represents the passage of FITC-dextran, was monitored by taking 40 μL medium aliquots over a time course to measure fluorescence using a SpectraMax microplate reader (SpectraMax M5, USA) at 520 nm.

For the trans-endothelial migration assay, pHIMECs were grown to form a monolayer in the upper well of a 24-well Transwell plate (8 μm pore size; BD Biosciences), and PBS or *E. lenta* (MOI of 1:1000) was added to the culture. After incubation for 24 h, HCT-116 cells (1 × 10^4^) were added, and the transmigrated HCT-116 cells in the bottom wells were fixed with methanol and stained with crystal violet after 24 h of incubation. Each experiment was repeated six times, and each time three wells were tested for each sample.

### ATP viability assay

ATP viability assays were performed with a CellTiter-Glo® 2.0 assay for cell lines according to the manufacturer’s protocol (Promega, WI, USA). Cells were lysed with CellTiter-Glo® Reagent (100 µL) for 30 min. Luminescence signals were detected with a microplate reader (BioTek, VT, USA).

### Annexin V/propidium iodide (PI) apoptosis assay

Cells were cultured in 6-well plates with PBS, DH5α, or *E. lenta* alone, in combination with si-NC or in combination with si-PERK for 24 h. The cells were stained with FITC-annexin V solution and PI solution before being examined using flow cytometry. The number of positive cells was determined by summing the number of cells that were Annexin V+/PI+, Annexin V+/PI−, and Annexin V–/PI+.

### Metagenomic sequencing

Fecal DNA was extracted using the QIAamp DNA Stool Mini Kit (Qiagen, Hilden, Germany). DNA integrity, size, and concentration were determined by agarose gel electrophoresis and NanoDrop spectrophotometry (NanoDrop, Germany). After the sequencing libraries were constructed and quality controlled, high-throughput sequencing was performed using the NovaSeq 6000 platform (Illumina).

Raw sequencing reads were processed to obtain valid reads as previously described^24^. Quality-filtered reads were obtained and reassembled using IDBA-UD (V1.1.1). The clean reads were aligned to the database (V202003, (ftp://ftp.ccb.jhu.edu/pub/data/kraken2_dbs/)) using Kraken2 software (V2.1.1) to obtain species-level information for further analysis.

### scRNA-seq

For scRNA-seq, fresh samples were harvested immediately and prepared into single-cell suspensions. Dead cells were removed to improve the viability of the samples. Cells were loaded onto the 10X Chromium Single-Cell Platform (10X Genomics) at a concentration of 1000 cells/μL (Single-Cell 3′ library and Gel Bead Kit v3). The generation of gel beads in emulsion (GEMs), barcoding, GEM-RT clean-up, complementary DNA amplification, and library construction were performed following the manufacturer’s protocol. The final library pool was sequenced on an Illumina NovaSeq 6000.

#### Bioinformatic alignment and generation of the data matrix

For droplet-based scRNA-seq, sequenced fastq files were aligned, filtered, and barcoded, and unique molecular identifiers (UMIs) were counted using CellRanger Chromium Single-Cell RNA-seq version 2.0.2 (10X Genomics) and a custom reference package for the human reference genome Ensembl93 (GRCh38). Finally, a filtered matrix was used to generate Seurat objects for each sample using the Seurat (v5.1.0) workflow. The Seurat objects were subsequently merged and used for downstream analysis.

#### Unbiased clustering and nomination

For cell type identification, lineage-specific markers were used to distinguish cells at the major cell type level. The expression of cluster-specific marker genes was determined by performing differential gene expression analysis between cells from the individual cluster and cells from all other clusters using FindAllMarkers in Seurat with the following parameters: min.pct: 0.1, logfc.threshold: 1.0, and only.pos: TRUE. Only genes with a significant adjusted *P* value (*P* < 0.05, FDR-adjusted *P* value) were retained to define major cell types, including epithelial cells, endothelial cells, T/NK cells, B cells, plasma cells, fibroblasts, neutrophils, myeloid cells, mast cells, tuft cells, and mucosal enteric glial cells. The expression of markers from each cell type, such as EPCAM, PECAM1, CD3D, MS4A1, and MZB1, in the corresponding clusters was further confirmed and visualized using Vlnplot in Seurat.

#### Differential gene expression and pathway enrichment analysis

The DEGs identified in the two subgroups were evaluated using FindMarker with the following parameters: min.pct: 0.1 and logfc.threshold: 0. Genes with abs(avg_log_2_FC) > 0.5 and *P* value < 0.05 were termed significantly up- or downregulated genes. To determine the functional annotation of the significantly expressed genes and predict potential signaling pathways, gene enrichment analysis was performed on the basis of an ontology gene set (C5) (previously GO gene sets) downloaded from the Molecular Signatures Database (MSigDB, v2022.1. Mm), a joint project of UC San Diego and Broad Institute^25^. Genes in the output list were annotated using ENTREZID or ENSEMBL based on “org.Hs.eg.db” (v3.19.1) and then ranked in descending order of their average expression. The well-arranged gene list was used as input for pathway enrichment analysis using the enrichGO function in clusterProfiler (v4.12.0). The significantly different relevant GO pathways (cutoff value: *P* = 0.05, abs(NES) = 1) were visualized using ggplot2 (v3.5.1).

### Adhesion assays

All equipment, solutions, and plates used to cultivate anaerobic bacteria were pre-incubated in an anaerobic chamber one day prior to the adhesion experiment. *E. coli* DH5α and *E. lenta* strains that had been cultured for 48 h were resuspended in 1.5 mL of PBS-Ca/Mg. The 12-well Transwell plate with the pHIMEC monolayer was incubated with bacteria for 4 h under anaerobic conditions (80% N_2_, 10% CO_2_, and 10% H_2_). After incubation, the plate was washed twice with PBS-Ca/Mg, and the pHIMEC monolayer, which potentially had bacteria bound to the surface, was detached with 300 μL of a 0.1% trypsin solution for 5 min at 37 °C under anaerobic conditions. These wells were further washed two times with PBS-Ca/Mg followed by centrifugation at 7500× *g* for 5 min. The pellet was then resuspended in 1 mL of PBS Ca/Mg and serially diluted in PBS without Ca/Mg using a 10-fold dilution, which was followed by plating onto Wilkins–Chalgren agar with L-cysteine hydrochloride monohydrate and resazurin to enumerate the bacteria.

### Immunofluorescence

IF analysis of FFPE colon tumor/healthy colon tissue sections was performed. Three-millimeter-thick sections were prepared from FFPE human CRC and healthy control tissue blocks, deparaffinized, rehydrated, and blocked with 0.1 M Tris-HCl, pH 7.4, 2% fetal bovine serum, and 0.3% Triton X-100. The sections were then stained with the following antibodies: anti-PV-1 (Abcam, #27853; #321889, 1 µg/mL), anti-CD34 (Abcam, #81289, 1:100), or anti-ZO-1 (Abcam, #307799, 1:500). The sections were incubated with primary antibodies at 4 °C. The sections were then incubated with the appropriate fluorophore-conjugated secondary antibody. Before imaging, the nuclei were counterstained with DAPI. One drop of 50% glycerin was added to each section, and laser confocal microscopy (Nikon, Japan) was used to detect PV-1, CD34, and ZO-1 expression. Fiji software package was used for image analysis and fluorescence quantification.

For IF staining, the pHIMECs were co-cultured with PBS or *E. lenta* (MOI of 1:1000) in complete medium for 24 h. Then, the cells were fixed with 4% paraformaldehyde in PBS supplemented with 0.2% Triton. The cells were then blocked for 1 h with 1% bovine serum albumin, followed by incubation with an anti-vinculin antibody (Abcam, #129002, 1:100) or an anti- ZO-1 antibody (Abcam, #307799, 1:500) overnight at 4 °C. The cells were subsequently washed and incubated with the appropriate secondary antibody (Abcam) and DAPI.

For immunohistochemical staining, mouse liver tissues were fixed in 4% paraformaldehyde, embedded in paraffin, cut into 3 µm-thick sections, and stained with H&E according to standard protocols.

### FISH

FISH of colon tumors was performed on FFPE blocks. The tissue sections were subsequently washed in 0.1 M Tris-HCl, pH 7.4, for 15 min, after which the Gram-positive bacterial cell walls were hydrolyzed using lysozyme (10 mg/mL) for 30 min at 37 °C. The tissue sections were then hybridized with a 5ʹ Cy3-labeled EUB338 probe (5ʹ-GCTGCCTCCCGTAGGAGT-3ʹ)^26^ or an *E. lenta* probe (5ʹ-CCTTGCCGTCTGGGCTTT-3ʹ)^27^ in hybridization buffer containing 0.9 M NaCl, 0.02 M Tris-HCl, pH 7.4, and 0.01% SDS at 50 °C overnight. All probes were acquired from probeBase (http://www.microbialecology.net/probebase/) and synthesized by Sangon Biotech Company (Shanghai, China).

### Western blot

Western blot assays of the cells were performed following a previously reported method^28^. The primary antibodies used in the present study included anti-BiP antibody (Cell Signaling Technology, #3177, 1:1000), anti-phospho-PERK antibody (Cell Signaling Technology, #3179, 1:1000), anti-PERK antibody (Cell Signaling Technology, #3192, 1:1000), anti-phospho-eIF2α antibody (Cell Signaling Technology, #3398, 1:1000), anti-eIF2α antibody (Cell Signaling Technology, #5324, 1:1000), anti-ATF-4 antibody (Cell Signaling Technology, #11815, 1:1000), anti-ZO-1 antibody (Cell Signaling Technology, #8193, 1:1000), anti-Claudin-5 antibody (Cell Signaling Technology, #49564, 1:1000), anti-SQSTM1/p62 antibody (Cell Signaling Technology, #88588, 1:1000), anti-LC3A/B antibody (Cell Signaling Technology, #88589, 1:1000), anti-Beclin-1 antibody (Cell Signaling Technology, #3495, 1:1000), anti-Toll-like Receptor 4 antibody (Cell Signaling Technology, #38519, 1:1000), anti-GAPDH antibody (Abcam, #8245, 1:500). The antigen–antibody complex on the membrane was detected with enhanced chemiluminescence reagents (Thermo Scientific, Waltham, USA).

### qPCR

qPCR analyses of mouse colon tumor tissues were conducted following previously described protocols^29^. All the experiments were performed in triplicate, and β-actin was selected as the reference gene. Relative gene expression levels were calculated using the 2^–ΔΔCT^ method. The sequences of the specific primers used are listed in Supplementary Table S10.

### Electron microscopy

For transmission electron microscopy (TEM) analysis, the cells were collected and immediately fixed overnight in 3% glutaraldehyde. Afterward, the samples were rinsed three times with PBS and postfixed with 1% osmic acid for 2 h. After being rinsed three times with deionized water and serially dehydrated with 50%, 70%, 80%, 90%, and 100% alcohol and 100% acetone, the samples were embedded in epoxy resin to form blocks of cells. Ultrathin sections (50 nm) were obtained with an ultramicrotome (Ultracut UCT, Leica, Germany). The sections were then stained with lead citrate and uranyl acetate and examined by TEM (T10, FEI, USA).

For SEM analysis, after treatment with *E. lenta*, the cells were fixed with glutaraldehyde for 2 h at room temperature and stored at 4 °C. The samples were then rinsed three times with PB. Secondary fixation was performed using 1% osmium tetroxide in phosphate buffer (PB) for 1–2 h, followed by three additional PB washes. Dehydration was performed through an ethanol gradient (30%–100%), with critical point drying. The samples were sputter-coated with a gold layer and imaged under a scanning electron microscope (Hitachi, SU8100).

### Statistical analysis

Differences in the quantitative data between two groups were performed using the unpaired two-tailed Student’s *t*-test or Mann–Whitney *U* test, where appropriate. Comparisons of means among multiple groups were determined by one-way ANOVA followed by Tukey’s post hoc comparison test. The relationships between the abundance of *E. lenta* and the MFI of PV-1 relative to CD34 were analyzed by using two-tailed nonparametric Spearman correlation and linear regression. The associations between the patient categorical characteristics were analyzed using Fisher’s exact test. Overall survival was defined as the time interval from surgery to the date of death or the last follow-up. The survival curves and early recurrence curves were constructed using Kaplan–Meier model, and log-rank tests were used to determine the statistical difference. *P* values < 0.05 were considered significantly different (*P < 0.05, **P < 0.01, ***P < 0.001). All statistical analyses were performed using GraphPad Prism 8 software (GraphPad lnc.) or IBM SPSS Statistics 20.0 software (IBM lnc., SPSS).

## Supplementary information

Supplementary information

Supplementary Table S1

Supplementary Table S2

Supplementary Table S3

Supplementary Table S4

Supplementary Table S5

Supplementary Table S6

Supplementary Table S7

Supplementary Table S8

Supplementary Table S9

Supplementary Table S10

Supplementary Table S11
