# Supplementary material for: Eggerthella lenta disrupts the gut vascular barrier to drive colorectal cancer liver metastasis
Source: Cell Discov. 2026 Sep 22;12:67. doi: 10.1038/s41421-026-00922-4 (PMC13594196; doi:10.1038/s41421-026-00922-4)
Supplement: Supplementary file 1 [file 41421_2026_922_MOESM1_ESM.pdf]

Supplementary Fig. S1

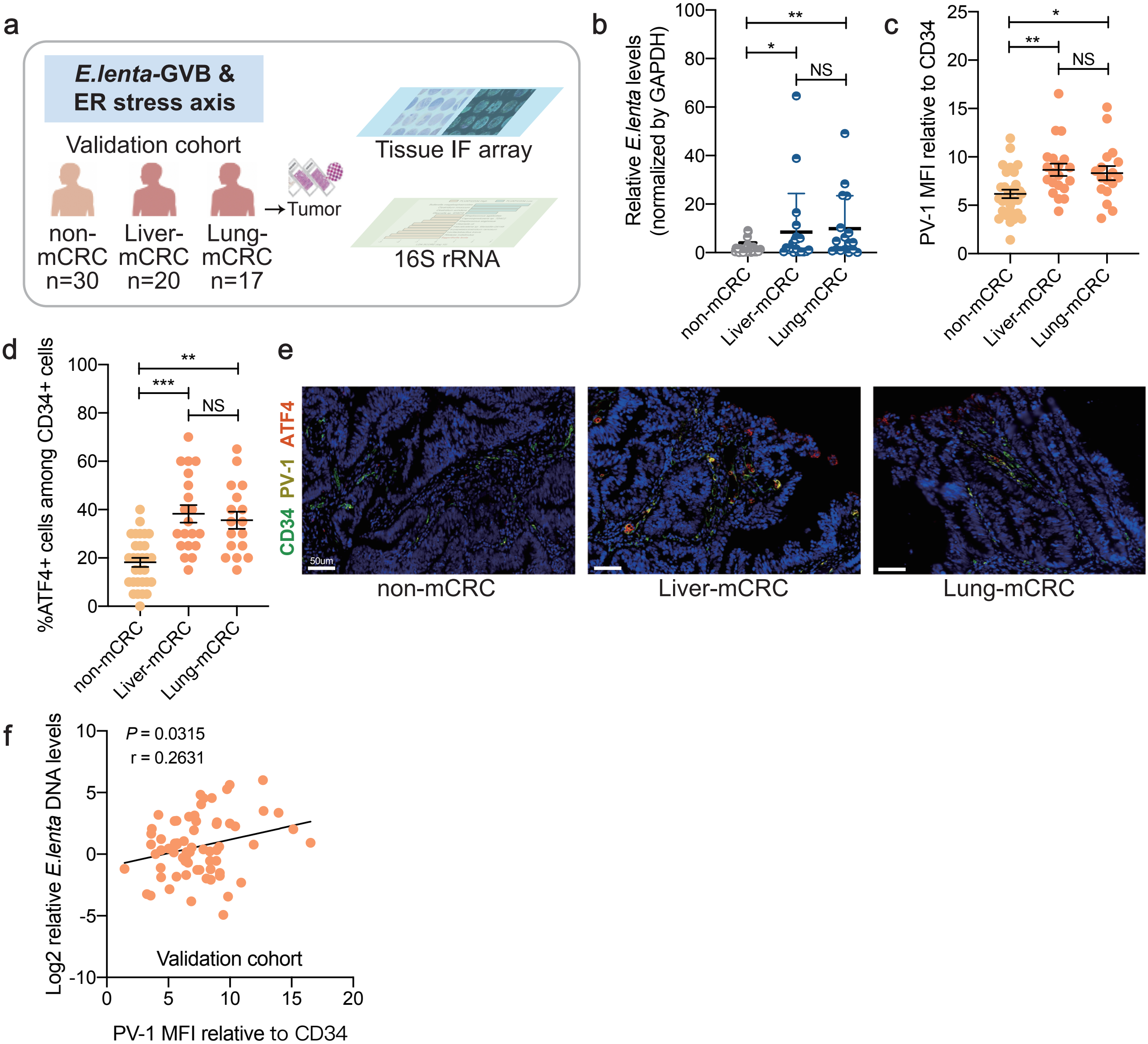

Supplementary Fig. S1. Validation cohort analysis reveals E. lenta prevalence and organ-specific metastatic susceptibility

(a) Schematic diagram depicting the design of tumor tissue immunofluorescence (IF) staining and 16S rRNA sequencing in the FUSCC validation cohort (non-metastases CRC, n=30; CRC liver metastases, n=20; CRC lung metastases, n=17) to identify GVB damage heterogeneity and ER stress.
(b) Relative E.lenta levels in tumor tissues detected by 16S rRNA sequencing.
(c) Quantify the mean fluorescent intensity (MFI) of PV-1 among the CD34 positive vessels in tumors.
(d) Percentage of ATF4 positive cells among CD34 positive cells in tumors.
(e) Representative images of CD34, PV-1, and ATF4 IF staining in tumors.
(f) The correlation of tumor vessel PV-1 expression and tumor E. lenta levels in all patients from the FUSCC validation cohort (n = 67; $P < 0.05$ by two-tailed nonparametric Spearman correlation; linear regression).
(b-d) Data are represented as mean ± SEM using scatter dot plots. Statistical significance was evaluated using one-way ANOVA followed by Tukey's post-hoc comparison test. **P* < 0.05; ***P* < 0.01; ****P* < 0.001.

Supplementary Fig. S2

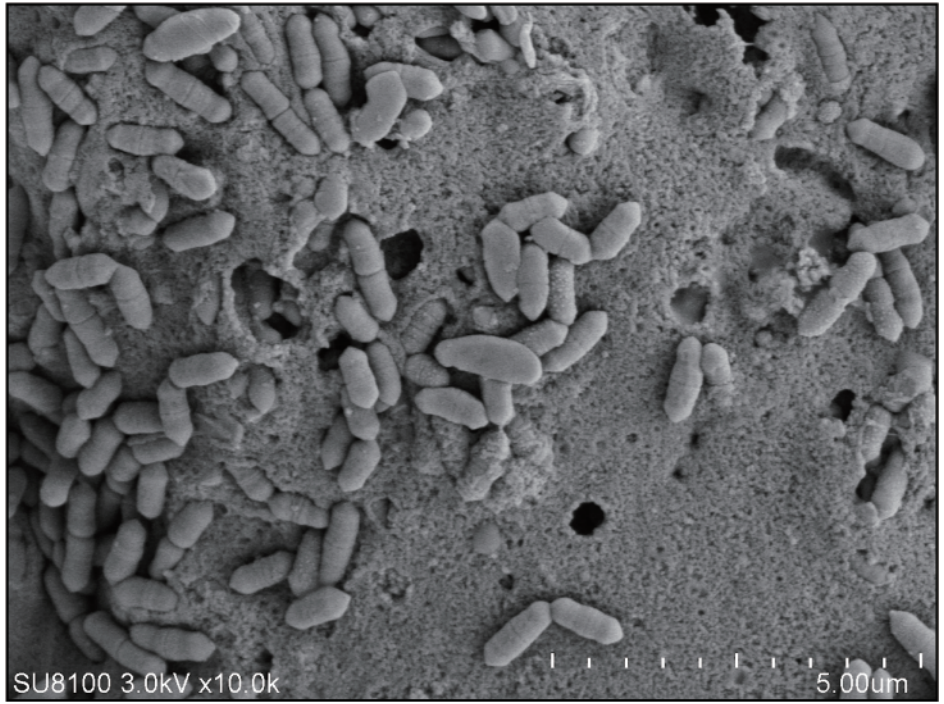

1:10 dilution

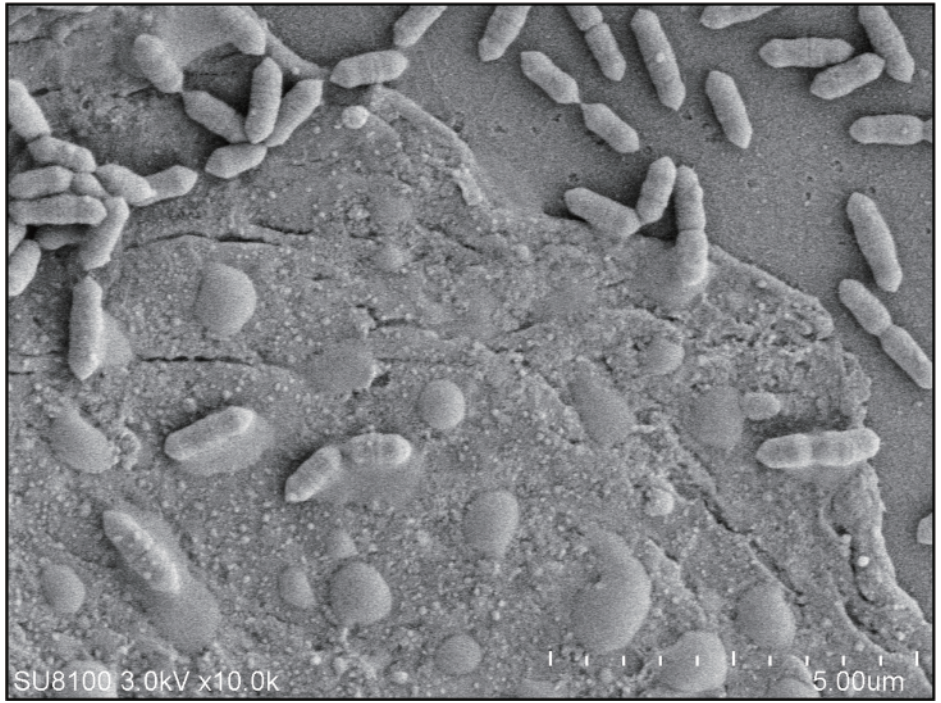

1:100 dilution

Supplementary Fig. S2. Scanning electron microscopy (SEM) visualization of E. lenta adhesion to human intestinal microvascular endothelial cells (HIMECs) under differential dilutions (1:10 and 1:100).

Supplementary Fig. S3

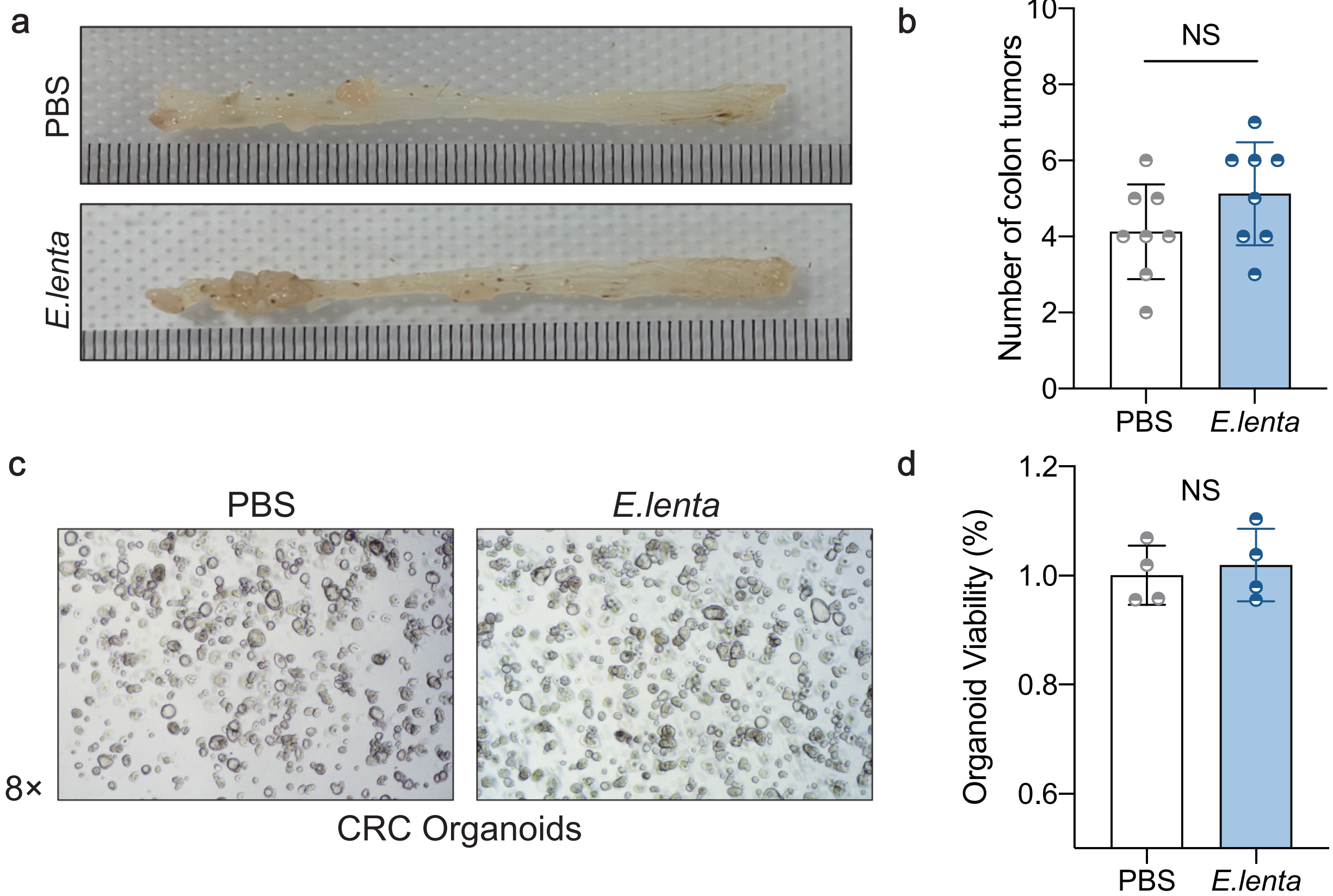

Supplementary Fig. S3. Impact of E.lenta administration on colorectal cancer tumor growth.
(a) Representative images of harvested colon tumors in mice treated with E. lenta or PBS.
(b) Quantification of colon tumors in mice (n = 8 per group).
(c-d) Colorectal cancer organoid viability was assessed by ATP assay after incubation with E. lenta or PBS (n = 4 per group).
(b and d) Data are represented as mean ± SD using scatter dot plots. Statistical significance was evaluated using two-sided Mann-Whitney unpaired test.

Supplementary Fig. S4

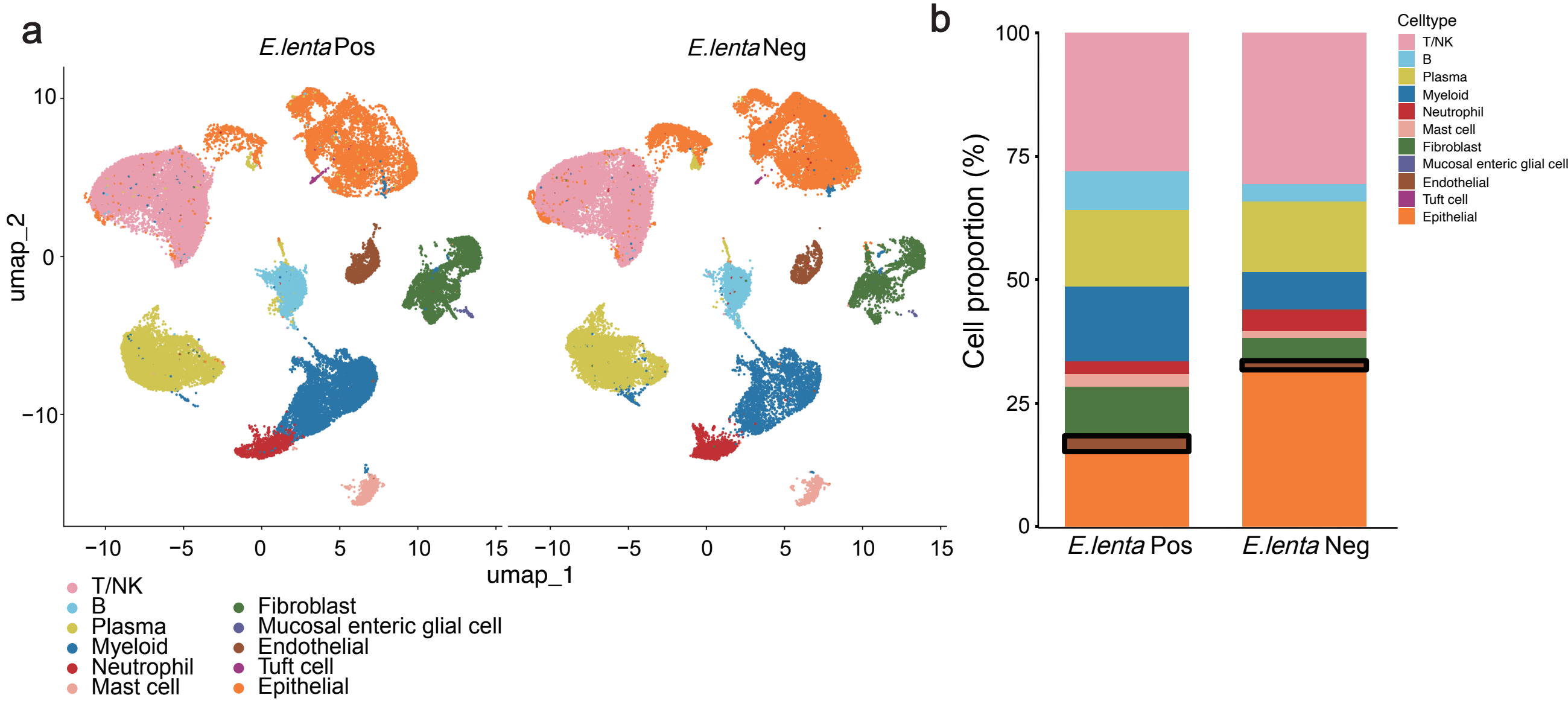

Supplementary Fig. S4. The single-cell sequencing analysis of total cells.

(a) The UMAP plots showing cell types of total cells from five E. lenta positive CRC tissues and five E. lenta negative CRC tissues.

(b) Percentage bar chart of the cell proportion in both groups.

Supplementary Fig. S5

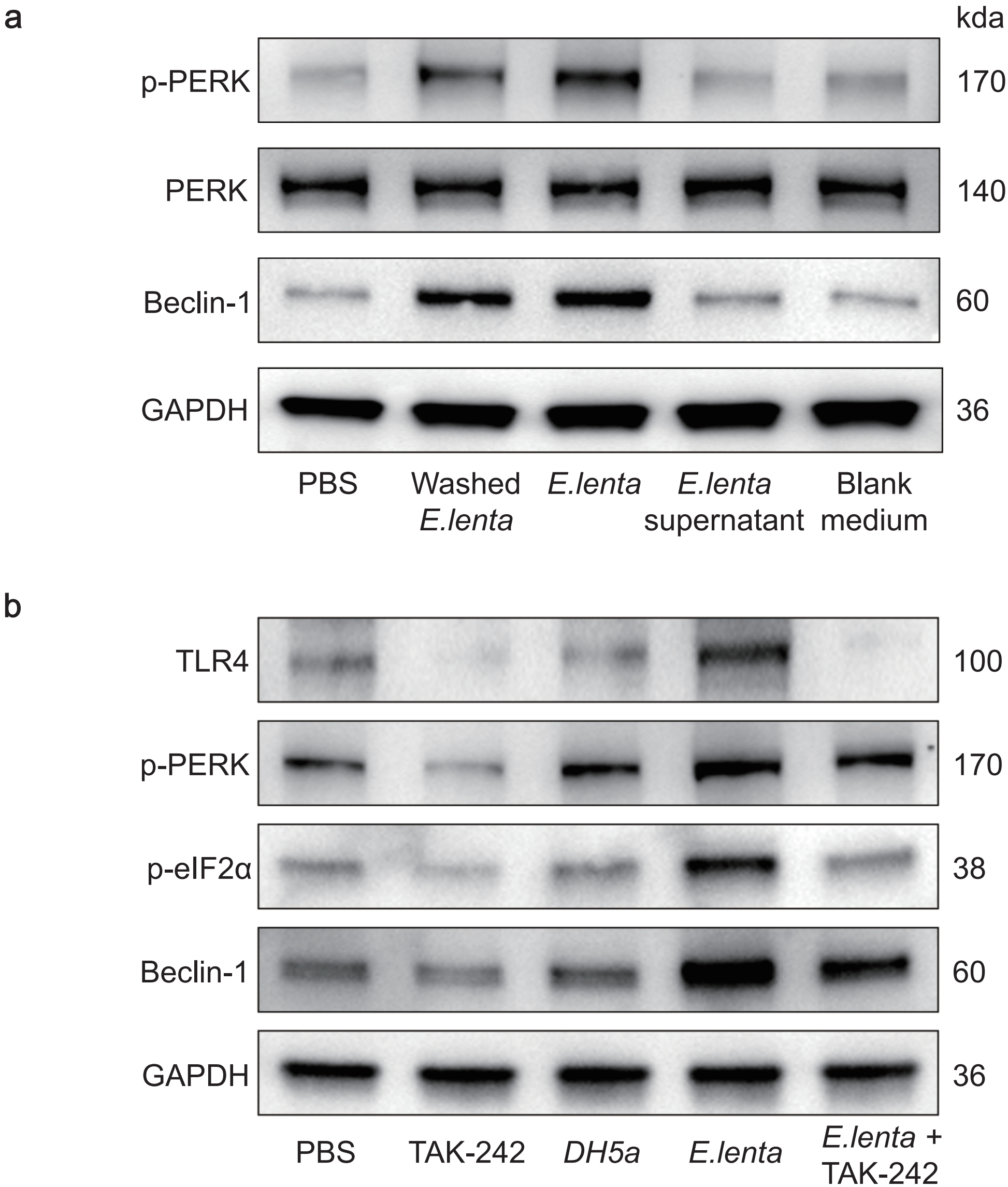

Supplementary Fig. S5. E. lenta surface proteins induce pHIMEC ER stress via TLR4-dependent signaling cascade.

(a) pHIMECs were treated with multiple washed E. lenta or E. lenta culture supernatant, and then analyzed by western blot for the protein level of ER stress and autophagy markers.

(b) pHIMECs were treated with TLR4 specific inhibitor TAK-242, DH5α, or E. lenta along with TAK-242, and then analyzed by western blot for the protein level of TLR4, ER stress and autophagy markers.

Supplementary Fig. S6

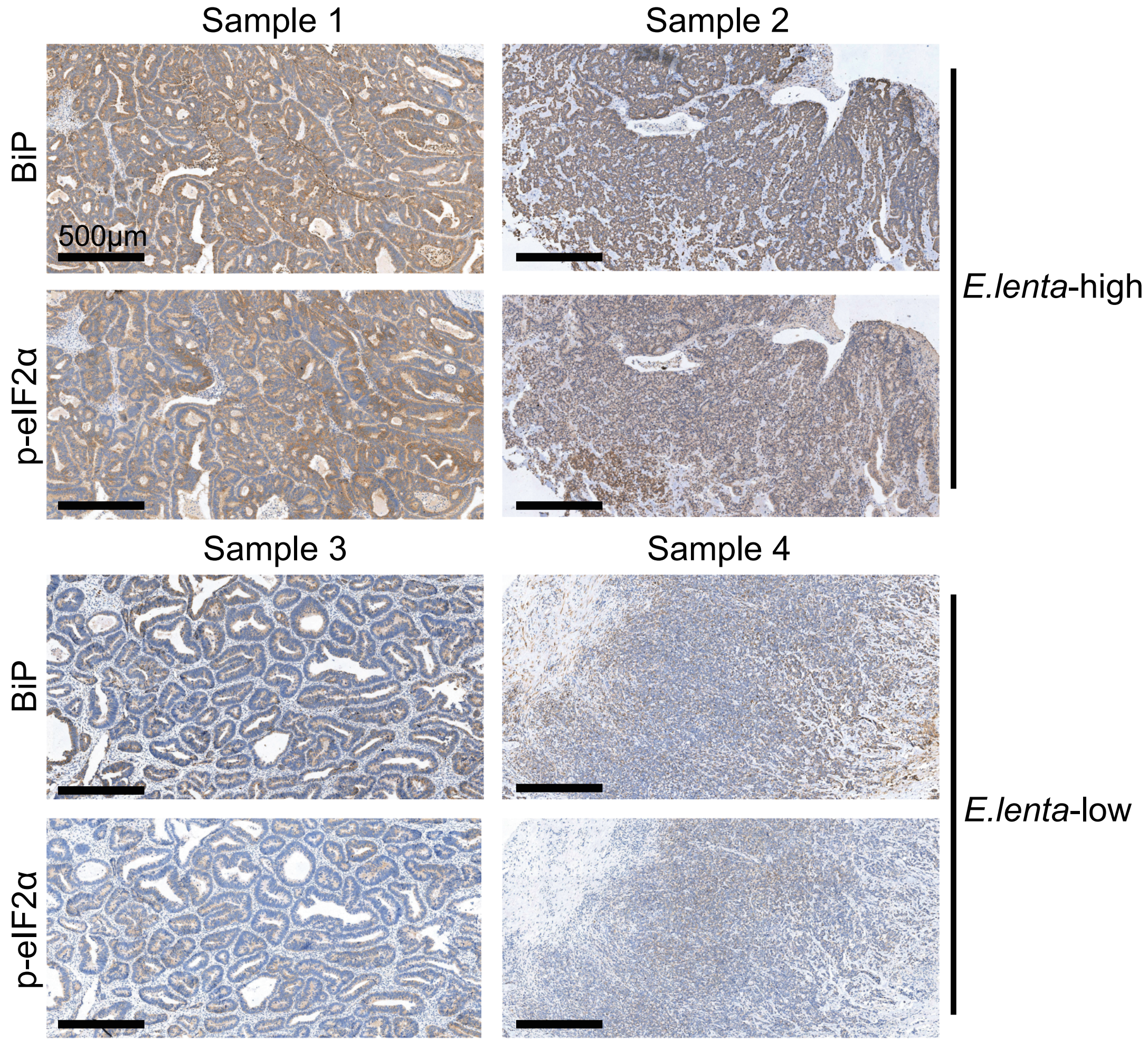

Supplementary Fig. S6. Representative immunohistochemical staining images of BiP and p-eIF2α in E. lenta-positive and E. lenta-negative human CRC tissues.
